# Barriers and Facilitators to Participation in Psychosocial Research for Adults With Intellectual Disabilities: A Systematic Review

**DOI:** 10.1111/jar.70267

**Published:** 2026-07-21

**Authors:** Kirsty Firman, Katie Bigham, Christopher Saville, Elizabeth Burnside

**Affiliations:** ^1^ North Wales Clinical Psychology Programme, Bangor University Bangor UK; ^2^ Learning Disabilities Team, Betsi Cadwaladr University Health Board Bangor UK

**Keywords:** barriers, facilitators, intellectual disabilities, recruitment, research participation, systematic review

## Abstract

**Background:**

People with intellectual disabilities often experience challenges to participation in psychosocial research. This review examined the barriers and facilitators affecting their participation.

**Methods:**

Preferred Reporting Items for Systematic Reviews and Meta‐Analyses (PRISMA) guidelines were followed. Databases searched included: PsycINFO, PubMed, Web of Science, and Scopus. A narrative synthesis structured using the Socio‐Ecological Model, considering levels of influence—intrapersonal, interpersonal, institutional, community, and policy—was used.

**Results:**

There were specific and shared barriers and facilitators for different levels of influence involved in psychosocial research. Contradictions between levels of influence, including beliefs about attitudes, views around harms, and the use of incentives, are discussed.

**Conclusions:**

The importance of targeting levels of influence utilising different methods to improve the participation of adults with intellectual disabilities in psychosocial research is highlighted.

## Lay Summary

- This research aimed to understand the challenges for adults with intellectual disabilities to participate in psychosocial research and what can help with this.
- There are different levels of influence involved in psychosocial research, including adults with intellectual disabilities; their family and carers; institutions—support services, researchers, and ethics committees; community—the relationship between institutions and other groups; and policies. This has an impact on participation in research.
- For levels of influence, there are different and shared factors that impede and help adults with intellectual disabilities to participate.
- Researchers should work with all levels of influence in psychosocial research to help adults with intellectual disabilities participate.

## 1 Introduction

People with intellectual disabilities have the same rights as those without intellectual disabilities (United Nations Convention on the Rights of Persons with Disabilities [CRPD] 2006). These rights include being able to make their own decisions and fully enjoy all human rights and fundamental freedoms (CRPD 2006). Consequently, these rights support the principle that people with intellectual disabilities should be included in research on an equal basis with others, with appropriate supports where required, and should not be excluded without ethical justification (Association of Medical Research Charities 2022; CRPD 2006; United Nations Human Rights Council 2024).

Guidelines to support the inclusion of people with intellectual disabilities in research are established both internationally and nationally. Internationally, the need for special protections, gatekeeper engagement, and respecting assent and dissent is highlighted (Council for International Organizations of Medical Sciences 2016; World Medical Association 2024). In the United Kingdom (UK), Department of Health (2005) states that capacity is assumed unless proven otherwise; decision‐making should be supported, such as through adapted resources; and decisions are made in the person's best interests if they lack capacity. Additional frameworks and guidance emphasise the importance of making research more inclusive. This includes using easy‐read materials, co‐researcher roles, and adapting consent processes to increase accessibility (Equality Act 2010; National Institute for Health and Care Research [NIHR] 2020).

Despite these frameworks, people with intellectual disabilities remain underrepresented in research, particularly as research participants—individuals who contribute data (Brooker et al. 2015; Feldman et al. 2014; Russell et al. 2019; Shankar et al. 2018). Research demonstrates how many studies exclude these individuals at initial stages of study design and participant recruitment (McDonald et al. 2022; Shepherd et al. 2019; Spaul et al. 2020). Exclusion commonly occurs across both biomedical and psychosocial research, and across a range of qualitative and quantitative methodologies, such as clinical trials, diagnostic/physiological studies, survey‐based studies, interviews, and focus groups. People with intellectual disabilities are often directly excluded in the inclusion and exclusion criteria, often without consideration of individual cases (Brooker et al. 2015; Feldman et al. 2014; McDonald et al. 2022; Shepherd et al. 2019; Spaul et al. 2020). Alternatively, they may be indirectly excluded due to factors commonly associated with intellectual disability, including capacity to consent, recruitment methods (e.g., driver licence lists), high demands on participants, and difficulties reading or writing, with minimal accommodations made (Brooker et al. 2015; Feldman et al. 2014; McDonald et al. 2022). For studies that do not exclude them or actively seek to recruit them, there continue to be challenges regarding research participation, particularly with recruitment and data collection (Mulhall et al. 2018). Consequently, understanding barriers and facilitators to participation is crucial.

Psychosocial research generally concerns the relationship between an individual and their environment or context (Taylor and McAvoy 2015), and aims to understand the influence of social, psychological, and community factors on people's lives. Key areas of study include mental health, health behaviours (if exploring the impact of social and psychological factors on these), family and relationships, and the influence of community and society on functioning and well‐being (Larentis 2024). Such research commonly includes qualitative and quantitative methodologies, such as interviews, surveys, observational studies, ethnographic studies, and non‐pharmacological intervention studies. This also includes some forms of randomised controlled trials (RCTs) where outcomes are behavioural, psychological, or related to wellbeing rather than biological change; for example, evaluating the effectiveness of psychological or behavioural interventions.

People with intellectual disabilities frequently experience psychosocial challenges, which make understanding their experiences through research especially important. They are more likely to experience mental health difficulties (Mazza et al. 2020) and are also frequently excluded from meaningful and enjoyable activities that facilitate social connection (Dattilo 2013; Merrells et al. 2019). Such experiences contribute to loneliness, a significant predictor of poorer outcomes (Gilmore and Cuskelly 2014). Furthermore, people with intellectual disabilities have lower mental health service uptake rates and experience more barriers to accessing psychological support versus people without intellectual disabilities (Einfeld and Tonge 1996; Whittle et al. 2019). Despite psychosocial research rarely involving invasive procedures or direct biomedical risk, participation by people with intellectual disabilities remains challenging. This raises important questions about the barriers and facilitators to inclusion in psychosocial research.

People with intellectual disabilities also experience various physical health inequalities, including high rates of physical health conditions, compared to the general population (Cooper et al. 2015; Kinnear et al. 2018). Biomedical research can build understanding of this, and biomedical methodologies can occasionally be relevant to psychosocial research, such as trials of medication adjunct to talking therapies. However, distinct ethical and methodological issues exist for biomedical research. For this review, ‘biomedical research’ is defined as studies where either the independent or dependent variable is biological or physiological in nature. This includes clinical trials (e.g., drug trials or medical devices), observational biomedical studies (e.g., investigations of disease prevalence or biomarkers), and biomedical diagnostic procedures (e.g., laboratory tests including blood or urine tests, imaging studies, functional tests, or other diagnostic investigations, including diagnosis of physical health conditions). These studies often involve invasive procedures and increased perceived risk of harm (d'Abrera et al. 2013; Oliver‐Africano et al. 2010), which may contribute to anxiety and/or reluctance to participate among participants or stakeholders making decisions on their behalf (Cleaver et al. 2010; Oliver‐Africano et al. 2010). Consequently, these studies require increased ethical and regulatory scrutiny (World Medical Association 2024) and more complex consent processes (d'Abrera et al. 2013; Iacono and Murray 2003). Contrastingly, psychosocial research often only includes risks of more abstract forms of harm—threats to privacy and autonomy, and risk of distress—and so the barriers to participation will be different to those in biomedical research. Therefore, the present review focuses exclusively on psychosocial papers.

Several reviews have explored the inclusion of people with intellectual disabilities in research. Shariq et al. (2023) investigated recruitment barriers and facilitators for clinical trials by disabled people, including those with intellectual disabilities. Identified themes include risk versus benefit assessment, design and management of recruitment protocol, consent and ethics, and systemic factors. Mulhall et al. (2018) focused specifically on adults with intellectual disabilities participating in RCTs, highlighting challenges with recruitment, resistance to control groups, engaging with carers, staff, and stakeholders, and the need to adapt interventions and resources. McDonald et al. (2024) examined one specific barrier—consent—highlighting guiding principles, strategies to enhance consent, involving guardians, and strategies for expressing decisions and enhancing ongoing decisions. While these reviews provide important considerations, they are limited in scope, either focusing on clinical trials or single barriers. The present review extends this body of knowledge by synthesising evidence for a wide range of barriers and facilitators across a range of psychosocial research methodologies, rather than focusing on clinical trials or single barriers only. By doing this, the review aims to provide a broader and more comprehensive understanding of barriers and facilitators to participation in psychosocial research for people with intellectual disabilities, with implications for improving participation across a range of psychosocial research contexts.

Inclusive research and co‐production approaches, particularly those involving people with intellectual disabilities as co‐researchers, have been proposed as best practice for reducing barriers to participation (NIHR 2020). In these approaches, people with intellectual disabilities contribute to various research processes, including study design, recruitment, conduct, and dissemination (Nind and Vinha 2014). A systematic review by Di Lorito et al. (2018) examined barriers and facilitators to co‐research with adults with intellectual disabilities and their impact on those involved. While this literature is crucial and closely related to the topic of barriers and facilitators to participation in research, its main focus is on participation as co‐researchers rather than research participants. Nevertheless, Di Lorito et al. (2018) also emphasised how these approaches enhance research participation, such as through feelings of trust, safety, and being understood by people with lived experience. Therefore, although co‐production is not the central focus of the present review, studies highlighting its impact on participation will be included.

This systematic review therefore aimed to explore and synthesise existing literature investigating barriers and facilitators to participation in psychosocial research for adults with intellectual disabilities. The review aimed to answer the following questions:

1. What are the barriers to participation in psychosocial research for adults with intellectual disabilities?
2. What are the facilitators to participation in psychosocial research for adults with intellectual disabilities?

## 2 Methods

### 2.1 Protocol and Registration

This review was pre‐registered with the International Prospective Register of Systematic Reviews (PROSPERO; registration number CRD42024590459). Preferred Reporting Items for Systematic Reviews and Meta‐Analyses (PRISMA) guidance (Page et al. 2021) was used.

### 2.2 Search Strategy and Eligibility Criteria

A pre‐planned search of four databases—PsycINFO, PubMed, Web of Science, and Scopus—was conducted in July 2025. Scopus was used only to capture papers from the *Tizard Learning Disability Review* journal, which was not captured by the other databases. Terms used for the search strategy are shown in Table 1. Terms were developed based on knowledge of relevant literature and collaborative discussion with a specialist academic librarian and searched for within the title, keywords, and abstracts of papers. Condition‐specific terms, including Down syndrome and Fragile X syndrome, were included because they represent the most common genetic (Karam et al. 2015; Sherman et al. 2007) and inherited (McLennan et al. 2011; Saldarriaga et al. 2014) causes of intellectual disability, respectively, and are frequently used as proxies for intellectual disabilities in the literature. Including these terms ensured that papers referring only to these conditions, rather than the broader category of ‘intellectual disability’, were captured. Other, less common causes of intellectual disability were not included in the search as these are less frequently used in the literature and would be unlikely to capture additional relevant papers. Due to the review focusing on research participation, the term ‘co‐researcher’ was not included in the search strategy. However, articles examining the influence of co‐researchers with intellectual disabilities on research participation were eligible for inclusion where relevant.

**TABLE 1:** Search terms.

Intellectual disability	Participation in research	Barriers and/or facilitators
‘learning disabilit*’ OR ‘intellectual disabilit*’ OR ‘mental* retard*’ OR ‘down syndrome’ OR ‘down's syndrome’ OR ‘fragile X syndrome’	(stud* OR trial OR research) AND (participat* OR enrol* OR recruit* OR engage* OR involve*)	Challeng* OR reason* OR motivation* OR view* OR decision* OR attitude* OR willing* OR consideration* OR concern* OR barrier* OR issue* OR facilitat* OR refus* OR assent* OR consent* OR dissent* OR declin*

Search results were exported to Rayyan (Ouzzani et al. 2016). Duplicates were removed, first using the Rayyan automation tool and then manually. Title and abstract screening were then completed together. An initial sample of 20 articles was jointly screened at this stage by the first and third authors to clarify inclusion and exclusion criteria, which is summarised in Table 2. The remaining records were then independently screened by the first author. To ensure consistency and reliability, a 10% sample of these papers was independently screened by the third author.

**TABLE 2:** Inclusion and exclusion criteria.

Inclusion	Exclusion
Papers investigating adults (≥ 18 years) with an intellectual disability. Where papers include both children and adults, at least 75% of participants must be adults, or data for adults are reported separately.	Papers investigating children/adolescents with an intellectual disability, without separate adult data. Papers where it is unclear whether ≥ 75% of participants are adults.
Papers investigating barriers and facilitators to participation in research by adults with intellectual disabilities where these individuals are the focus of recruitment, consent, engagement, or involvement. Data may be collected from adults with intellectual disabilities themselves or from other stakeholders (e.g., researchers, service staff, parents) when it is specifically about participation by adults with intellectual disabilities.	Papers where the focus is not on participation by adults with intellectual disabilities. This includes papers where the focus is solely on barriers and facilitators to research participation by parents, caregivers, or proxies themselves, without reference to participation by adults with intellectual disabilities.
Papers in which at least 75% of participants are adults with an intellectual disability, including where an intellectual disability is present alongside other diagnoses (e.g., autism spectrum disorder).	Papers of other populations (e.g., autism, mental health, brain injury, dementia) where less than 75% of participants are adults with an intellectual disability, and separate analyses are not provided.
Papers investigating participation in psychosocial research (e.g., surveys, interviews, focus groups). This includes psychosocial aspects of health/biomedical research (e.g., health behaviours, public health interventions).	Papers of biomedical research participation involving studies where either the independent or dependent variable is biological or physiological in nature (e.g., drug/device trials, diagnostic procedures, observational biomedical studies).
Papers where the main focus is barriers and/or facilitators to research participation, including attitudes towards research.	Papers where barriers and/or facilitators are reported only secondarily or in passing (e.g., feasibility studies, brief reflections in primary papers).
Papers of participation by adults with intellectual disabilities as research participants, contributing data for the study.	Papers examining participation by adults with intellectual disabilities as co‐researchers—including in research design, analysis, and running studies only—without reference to participation as research participants.
Papers investigating how the involvement of co‐researchers with intellectual disabilities facilitates participation of adults with intellectual disabilities as research participants.	Papers investigating the experiences of adults with intellectual disabilities solely as co‐researchers, without specifically investigating the impact of their involvement on participation by other individuals with intellectual disabilities as research participants.
Empirical research papers (qualitative, quantitative, mixed methods) where data are collected and analysed.	Reflective, critical, or commentary papers that do not report primary data (e.g., opinion pieces, methodological reflections). Other systematic, scoping, or literature reviews.
Peer‐reviewed papers.	Non‐peer‐reviewed papers.
Papers published in English.	Papers not published in English.
Papers published within the last 20 years.	Papers not published within the last 20 years.

Full‐text reviews were then completed with the remaining articles to determine eligibility. The first author independently reviewed all full‐text articles. The fourth author independently reviewed a 20% sample. Reasons for full‐text sources not meeting inclusion criteria were recorded manually by the first author during full‐text screening. Citation and reference list searches were conducted manually on included papers from the main search to identify further relevant papers. If there was any uncertainty about whether identified papers should be included, this was discussed with the third author.

This review focused exclusively on participation in psychosocial research. Papers utilising biomedical methodologies were excluded. Papers investigating inclusive and co‐production research were included only if they explored the impact of this on research participation. This review also focused exclusively on adults with intellectual disabilities and excluded children and adolescents. While research participation for both adults and children or adolescents with intellectual disabilities involves significant complexities, the nature of these challenges is different. There are distinct legal and ethical frameworks for adults and children regarding consent to participate in research. For children and adolescents, parental or guardian consent is legally required for them to participate, and assent from the young person is sought when appropriate. In contrast, Department of Health (2005) emphasises that adults are assumed to have capacity to consent unless assessed otherwise. In these cases, they may be supported to make their own decisions. A parent or guardian will only be involved to provide consent when the individual lacks capacity. Because of these differences, inclusion of papers investigating children would introduce heterogeneity; therefore reducing the specificity and applicability of review findings to adults. Despite this, it is recognised that the challenges of participation are not always additional for children, and there are overlaps with the barriers and facilitators experienced by adults, particularly for older adolescents whose developmental stage may make their experiences of participation comparable to adults. This represents an important area for future systematic reviews.

### 2.3 Quality Appraisal

A quality appraisal was completed for all included papers using the Mixed Methods Appraisal Tool (MMAT; Hong et al. 2018). This appraisal tool was chosen since it was designed for systematic mixed studies reviews, fitting with the review's inclusion criteria. Appraisal criteria included clarity of research questions, appropriateness of methods, adequacy of data collection and analysis, consideration of limitations, and integration of methods for mixed methods papers. Quality appraisals for all included papers were conducted independently by the first and second authors who then discussed ratings. Disagreements on ratings were discussed, and decisions were agreed collaboratively. All papers were included in further analysis, regardless of their quality appraisal results. However, papers scoring higher were given greater weighting when addressing the research question. This was reflected in the reporting of the results section; findings from higher‐quality papers were given more emphasis, while lower‐quality papers were included but flagged to indicate caution in interpretation.

### 2.4 Data Extraction and Synthesis

Data to be extracted for data synthesis were pre‐determined and summarised into a summary table (see below). A synthesis of findings was then presented. Given the inclusion of quantitative, qualitative, and mixed methods papers, a narrative synthesis approach was used, following guidance from Popay et al. (2006).

A socio‐ecological framework was used to structure the synthesis. The socio‐ecological model (SEM; McLeroy et al. 1988) has previously been applied in the literature to examine barriers and facilitators to participation in research, including studies with minority and underserved groups (Elder et al. 2007; Pardhan et al. 2025; Salihu et al. 2015; Stockton et al. 2012; Wells and Zebrack 2008). It has also been applied to the intellectual disabilities population. For example, Simplican et al. (2015) applied the framework to understand social inclusion of people with intellectual disabilities, highlighting the importance of intervening at different levels of influence. The model has also been used in other studies investigating people with intellectual disabilities, including reviews (Khan et al. 2021; Sykes et al. 2024; Yu et al. 2022). These studies demonstrate the flexibility of the model for understanding complex information and support its suitability for synthesising findings in this review.

The SEM builds on Bronfenbrenner's (1974) ecological systems theory, which conceptualises human development as occurring within a complex system of relationships, from immediate relationships to broader cultural, political, economic, and social influences. The SEM adapts this model to focus on both individual and social‐environmental factors as targets for intervention, highlighting how multiple levels of influence impact behaviour and decision‐making (McLeroy et al. 1988).

**FIGURE 1:**
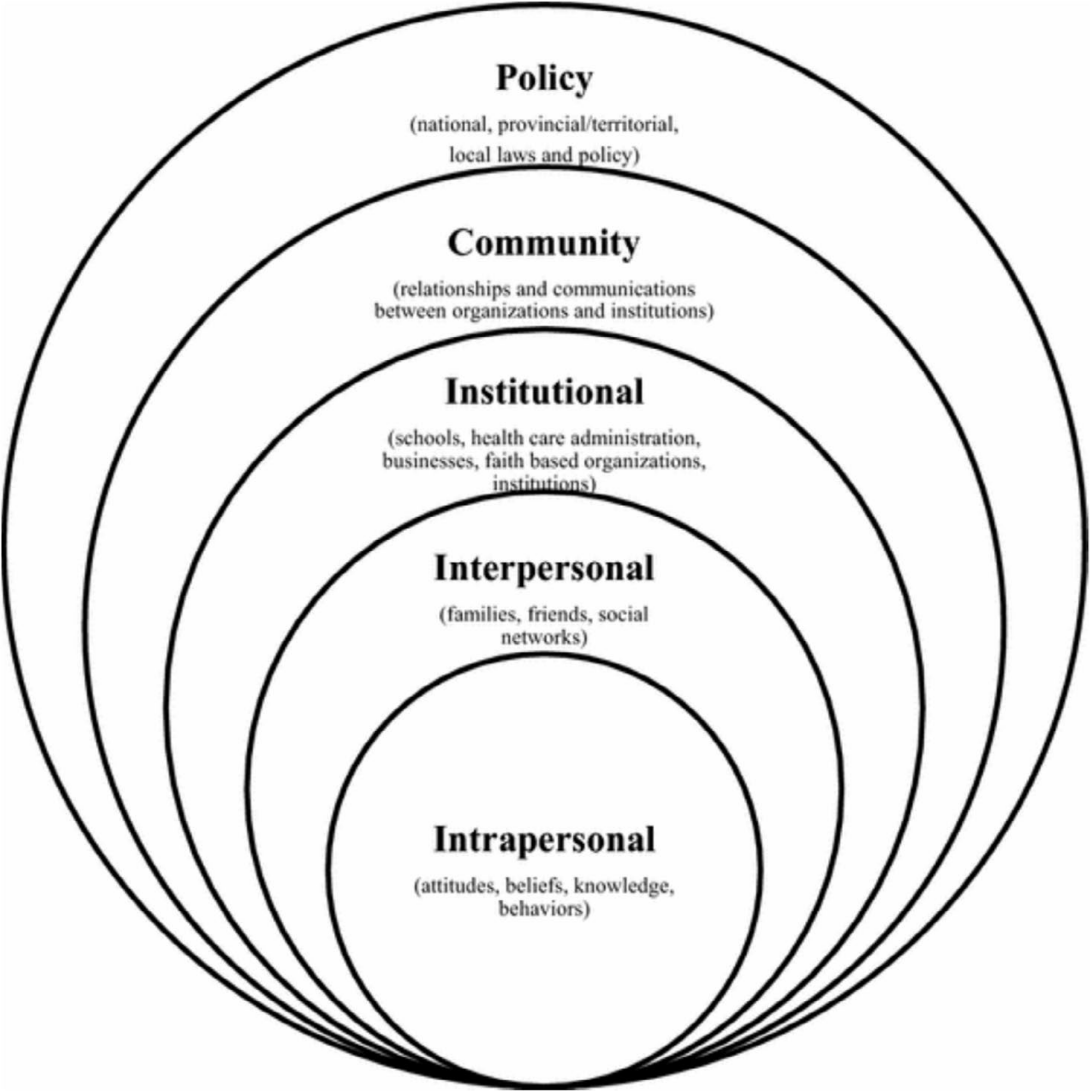
The socio‐ecological model.

As highlighted in Figure 1, the SEM identifies five levels of influence. Intrapersonal factors are characteristics of the individual, such as knowledge, attitudes, behaviours, self‐concept, and skills. Interpersonal processes reflect social networks and social support systems, including the family, carers, work groups, and friends. Institutional factors are organisations involved with individuals, such as schools, businesses, and services, including their influence on change. Community factors reflect relationships among organisations and groups within a certain area. Finally, public policy refers to policies, procedures, and laws that protect the community (McLeroy et al. 1988). These levels operate simultaneously within and between levels, subsequently impacting individual behaviour and the design of relevant interventions (Wells and Zebrack 2008). Factors identified in included papers were assigned to the level which best fit.

## 3 Results

### 3.1 Overview of Identified Papers

**FIGURE 2:**
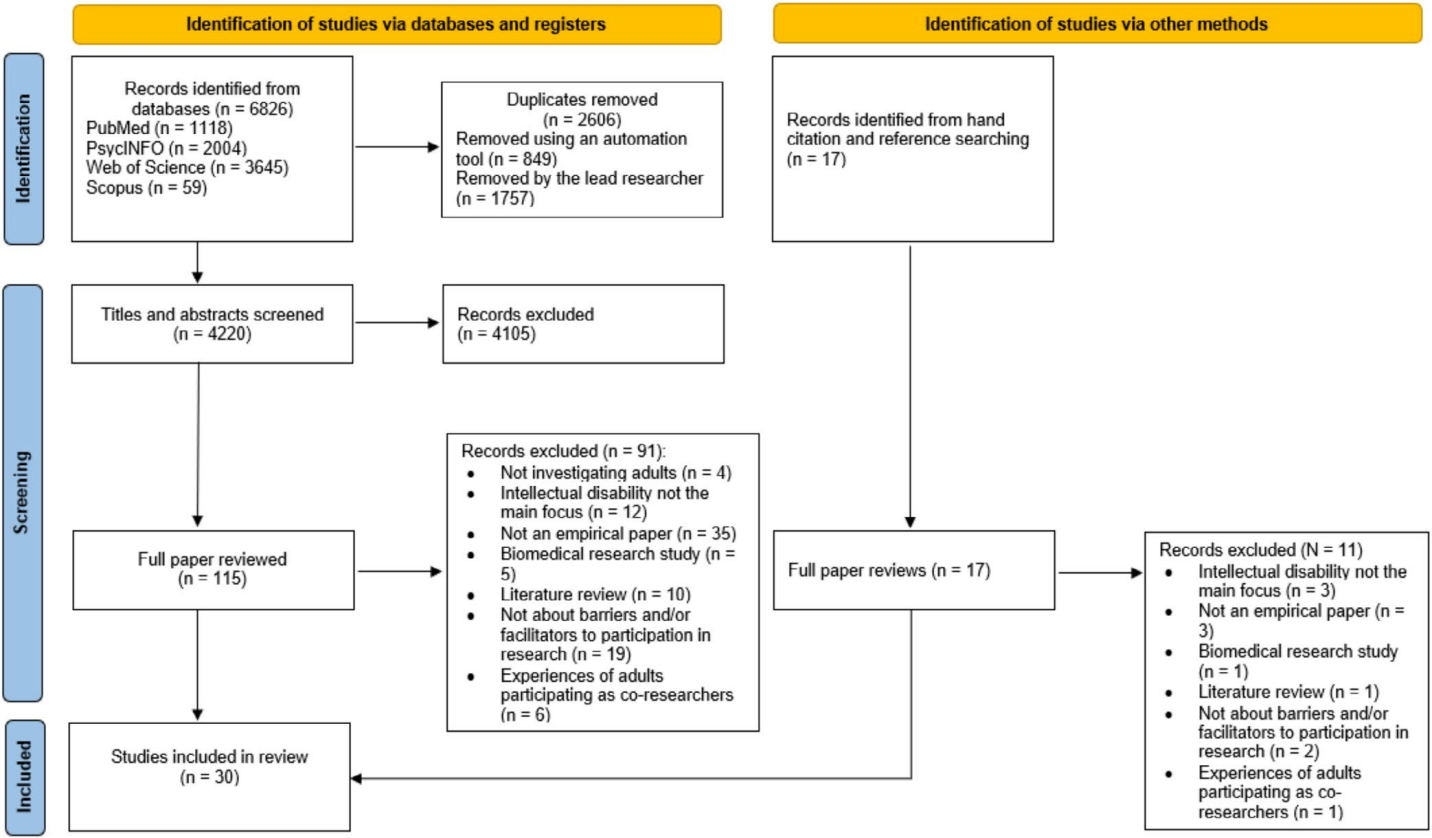
PRISMA diagram of selected papers.

As highlighted in Figure 2, initial searches returned 6826 papers. After removing duplicates, 4420 remained. Papers' titles and abstracts were screened, leaving 115 papers to retrieve. Following full paper review, 91 were excluded, leaving 24 to be included in the review. Citation and reference searches of included papers identified a further 17 possible papers. Following the same procedure, six additional papers met inclusion criteria. The final total of identified papers was therefore 30. Table 3 provides a summary of each paper.

**TABLE 3:** Summary of included papers.

Author(s), year, and title	Country	Type of study	Study design	Participants	Key findings
Bishop et al. (2024). The inclusion of adults with intellectual disabilities in health research—challenges, barriers and opportunities: A mixed‐methods study among stakeholders in England.	UK.	Mixed methods: quantitative descriptive (cross‐sectional) and qualitative (thematic analysis).	Stage 1: NIHR portfolio review and online survey investigating challenges and facilitators to inclusion for researchers of studies from the review. Stage 2: discussion of stage 1 findings in focus groups with experts‐by‐experience (EBE).	Stage 1: 52 researchers. Stage 2: 25 people with intellectual disabilities (comprised two EBE groups).	Stage 1: 77.7% of studies excluded participants with intellectual disability. Challenges included resource issues (23.5%) and perceived unsuitability (27.4%). Stage 2: Four main themes. (1) Research design and delivery—concerns about appropriateness; need for more planning and adaptations (e.g., easy‐read materials); perceived lack of researcher motivation and attitudes towards inclusion by EBEs; understanding the study, including researchers providing opportunities to ask questions; concerns of doing harm and liaising with gatekeepers; directly asking people about participation; recognising the positive aspects of inclusion. (2) Informed consent—ethics requirements seen as restrictive; challenges assessing capacity and consent; EBEs highlighted the need for accessible information, direct communication with participants, and use of consultees or legal representatives when needed. (3) Resources—need for increased funding, staff with relevant expertise and knowledge (including EBEs), and more time to design/deliver inclusive and accessible research. (4) Knowledge and skills—limited staff experience and knowledge in intellectual disabilities; need for training (research design, communication, reasonable adjustments), ideally delivered by people with intellectual disabilities.
Cameron and Murphy (2007). Obtaining consent to participate in research: The issue involved in including people with a range of learning and communication disabilities.	UK.	Quantitative non‐randomised and descriptive (cross‐sectional).	Data collected about participant recruitment and retention rates as part of a larger study. Data was also collected regarding consent through participants signing the consent form, researcher observations of positive indicators for giving consent, and subsequent discussion with carers.	48 adults with intellectual and communication disabilities.	It is important to provide accessible and understandable information as part of the recruitment and consent process. Face‐to‐face explanation is crucial to pick up both verbal and non‐verbal signals from participants—‘individualised communication’. There was a strong correlation between participants' assessed comprehension and their ability to give their own consent.
Conroy et al. (2021). A survey study of the attitudes and experiences of adults with intellectual disability regarding participation in research.	USA.	Quantitative non‐randomised and descriptive (cross‐sectional).	Survey of attitudes to research for adults with intellectual disabilities.	101 adults with intellectual disabilities.	Adults with intellectual disabilities generally believe they should have opportunities to participate directly in research and can make participation decisions with and without support. They hold positive views about research in general and are also generally trusting of researchers. Trust was related to more favourable views of research participation opportunities, general views of research, and interest in future research participation. The belief that research with adults with intellectual disabilities is important is a strong and unique predictor of willingness to participate in research.
Cook and Inglis (2009). Making our own decisions: Researching the process of ‘being informed’ with people with learning difficulties.	UK.	Qualitative (inductive analysis using NVivo; analytic method not reported).	Facilitated collaborative action research. Participants completed semi‐structured interviews investigating their understanding of research. They completed workshops designed to develop knowledge and understanding about research, to test methods supporting the process of informed consent, and to produce a training package for others potentially interested in research. Diaries of reflections were also kept by participants.	Seven men with intellectual disabilities (described as learning difficulties in the study but acknowledged as intellectual disabilities).	Three main themes. (1) What was already known about research?—there was some awareness of research but little understanding of meaning; participants knew they did not have to participate but could not explain why (2) Determining understanding—this was demonstrated when participants used their own words and ideas to explain; there was continued limited awareness of consequences of saying no to participating, especially when power dynamics were involved and factors impacted judgement of risks, for example, payment; metaphors and peer support aided understanding. (3) What supported the development of understanding—peer learning and support; repetition and recursiveness—having discussion time and asking further questions as well as repeating things; enjoyment and intellectual stimulation; simplifying language, including discussing the meaning of important words and using pictures.
Cook and Inglis (2012). Participatory research with men with learning disability: Informed consent.	UK.	Qualitative (use of NVivo; analysis method not reported).	Facilitated action research. All participants received information and consent forms in written and verbal form and were asked questions about participation. Attendance of workshops designed to promote discussion on topics related to research and included a DVD consisting of short scenarios about research. Participants were interviewed before and after workshops and were asked about their knowledge of research and what helped develop their knowledge. Reflective diaries were also kept by participants.	Seven men with intellectual disabilities (two withdrew). Staff (qualified and unqualified nurses) were also involved, but numbers were not reported.	Two main themes. (1) Illusory understanding—most men had previously participated in research and had some understanding of research, but there was varied understanding of concepts such as ‘interview’ and ‘data’, and whether ‘just talking’ could be research. Informed choice remained uncertain. (2) Constituents for learning—several approaches (DVDs, discussions, games, taking part in mock interviews, questionnaires, quizzes, and presenting their work to each other). The most effective approaches were: talking together, peer support, a recursive approach—revisiting ideas and asking questions again to deepen understanding—involving significant others, having fun, and using real examples.
Corby and Sweeney (2019). Researchers' experiences and lessons learned from doing mixed‐methods research with a population with intellectual disabilities: Insights from the SOPHIE study.	Ireland.	Mixed methods—qualitative (method unknown) and quantitative descriptive (cross‐sectional).	Phone calls to potential participants (adults with intellectual disabilities). Survey of researchers involved in the research. Questions about their experiences of participant recruitment and data collection, and reflections on any changes made based on experiences.	600 potential participants (adults with intellectual disabilities). Eight researchers.	Many potential participants did not participate because they were ‘not interested’ and because they did not have a family member to accompany them. It was necessary for researchers to have specialist training in intellectual disability when recruiting participants. Researchers were dependent on support from already busy service staff, and it was important for the research team to balance good response rates against becoming a burden or nuisance to staff members. The ability of the team to make contact directly by telephone made recruitment more likely. Being able to collect data outside of normal working hours is essential.
Crook et al. (2016). ‘So often they do not get recruited’: Exploring service user and staff perspectives on participation in learning disability research and the barriers that inhibit it.	UK.	Qualitative (thematic analysis).	Focus group with people with intellectual disabilities about their views and experiences of research participation. Survey to clinicians working with people with intellectual disabilities about their attitudes and beliefs about research participation, and perceived barriers.	Five adults with mild–moderate intellectual disabilities. 34 clinicians working with people with intellectual disabilities.	Research participation was important for people with intellectual disabilities and clinicians. Barriers to research participation can include gatekeepers' lack of understanding, time, and/or enthusiasm, and clinicians' concerns around capacity of people with intellectual disabilities to provide informed consent and/or that they may lack necessary skills to participate in research. The importance of promoting inclusive research approaches, producing accessible resources, and paying people for participation was highlighted.
Dye et al. (2007). Capacity of people with intellectual disability to consent to take part in a research study.	UK.	Quantitative RCT.	Consent information regarding taking part in an actual research project was presented to participants and their capacity was assessed using a questionnaire. Participants were assigned to three different ways of presenting passages about consent: control, section (reduced demand on memory), or photograph. Consent scores were analysed.	85 adults with mild intellectual disabilities.	There were no significant differences in scores for ability to consent between experimental conditions. The section and photograph conditions did not result in an increase in ability to consent. Only 5 participants were deemed able to consent. All participants could indicate a choice to participate, although only 69.4% demonstrated an understanding of the impact of choice.
Frankena et al. (2018). An exploration of the participation of people with intellectual disabilities in research—A structured interview survey.	The Netherlands.	Quantitative descriptive (cross‐sectional).	Structured interview survey about research participation, including frequency of participation, methods used, motivations to participate, and interests regarding study results.	508 adults with mild to moderate intellectual disabilities from the Panel Living Together (PLT) survey.	Nearly all respondents (92.7%) answered the question on their motivation to participate. Reasons included: expecting to enjoy it (73.5%) and finding research important (71.6%). For all motivations except ‘enjoy it’, the answer category ‘I don't know’ is relatively high. Only 11.8% of respondents indicated they were involved in research other than PLT surveys. 61% of respondents indicated an interest in the results of studies to which they contributed.
Hall et al. (2017). ‘…their opinions mean something’: Care staff's attitudes to health research involving people with intellectual disabilities.	UK.	Qualitative (thematic analysis).	Focus groups and telephone interviews exploring the attitudes of care staff who were not already involved in specific research projects involving people with intellectual disabilities. Exploration of barriers and potential solutions regarding recruitment and participation.	Eight employees of local care companies who were currently or had previously worked with people with intellectual disabilities.	Care staff felt that assisting their clients to participate in research could be a positive experience for both themselves and people with intellectual disabilities, if it was conducted in a way that was adapted and relevant for their clients. Barriers included: a lack of time and perceived benefits, people with intellectual disabilities finding it difficult to understand research demands, the consent process being difficult and time‐consuming, and organisational policies compromising research participation. Solutions included: appropriate planning and adaptations, flexibility when initially approaching care settings, support of management, and recognition of the key role of care staff.
Ho et al. (2018). Addressing challenges in gaining informed consent for a research study investigating falls in people with intellectual disability.	Australia.	Mixed methods—qualitative (case studies) and quantitative descriptive (cross‐sectional).	Data collected about participant recruitment and informed consent as part of a larger study.	68 older adults with intellectual disabilities for the initial approach about the research—recruitment data was collected for these participants. 40 for the informed consent process.	Just over 40% of caregivers declined on behalf of the person with intellectual disability due to illness, having ‘too much going on’, or believing the person with intellectual disability had nothing valuable to contribute to the study. Of the 40 participants enrolled, only three could successfully and independently answer the questions about the study and understood the potential risk. 22 participants, with their caregiver, met with the researcher about their involvement in the study. 15 did not engage with the researcher, and the consent process was completed without their involvement, by their caregiver instead.
Horner‐Johnson and Bailey (2013). Assessing understanding and obtaining consent from adults with intellectual disabilities for a health promotion study.	USA.	Quantitative descriptive (cross‐sectional).	Data collected about participant recruitment and informed consent as part of a larger study, including assessment of understanding about key aspects of study disclosures and understanding reasons for different responses and patterns of issues regarding understanding.	133 adults with mild to moderate intellectual disabilities.	More than half the sample were able to demonstrate adequate understanding of the study; 2 individuals were unable to answer any of the questions assessing understanding of the study protocol and were thus excluded, and 75 of the remaining 131 participants were able to answer all six questions. 43% of the sample had difficulty answering at least one question about the study, and 48% of participants had difficulty identifying potential risks of being in the study. Employing an authorised research representative provided an alternative to study exclusion.
Kyprianou et al. (2023). Caregivers' perception of adults with Down syndrome willingness to participate in research.	USA.	Quantitative non‐randomised and descriptive (cross‐sectional).	Online survey investigating caregiver interest in learning about future studies; perceived willingness of adults with Down syndrome to participate in different types of research, including surveys, observational studies, clinical tests, and lifestyle/technology; and demographic factors.	500 caregivers of adults with Down syndrome. 390 remained after excluding participants caring for individuals under the age of 18.	Only findings related to psychosocial research (surveys, exercise programmes, diet apps) were extracted. Responses relating to biomedical methodologies alone (blood draws, MRIs, drug/device trials, wearable‐tech monitoring) were outside the scope of the review. Most adults with Down syndrome had not previously participated in trials. Many caregivers (86% reporting yes or maybe) were interested in learning about future studies. Highest perceived willingness to participate was for non‐invasive studies (64.1% for surveys, 65% for memory tests) that could be conducted at home. There was also perceived high interest in trials involving tablets or phones, or daily exercise. Least willingness for invasive or time‐consuming trials. 14.1% would not ask the individual to participate. 57.7% reported that this was because they would not be interested. There was no significant effect of gender or caregiver education. Older adults with Down syndrome were less likely to have participated or be perceived as willing to participate.
Lennox et al. (2005). Beating the barriers: Recruitment of people with a learning disability to participate in research.	Australia.	Quantitative non‐randomised and descriptive (cross‐sectional).	Data collected about participant recruitment as part of a larger study, including recruitment success from three recruitment strategies: organisations distributing study information, telephone calls, and information sessions. Also structured interview questions on views on difficulties with recruitment and expectations of study involvement.	Initial hope to recruit 1000 adults with intellectual disabilities—recruitment data collected for these participants. 265 recruited and interviewed—interview data collected for these participants.	After doubling the length of time for recruitment, 265 out of 1000 participants had been recruited. Most participants were recruited following the implementation of the telephone strategy. 11% of participants consented on their own behalf, and 89% required another person to consent to their involvement. 75% of participants reported no difficulty enrolling, 7% reported specific difficulties with understanding what the study was about, and 6% reported difficulties completing consent forms. 36% said they expected to gain increased knowledge and awareness for themselves through participation, and 26% wanted to help others.
McDonald (2012). ‘We want respect’: Adults with intellectual and developmental disabilities address respect in research.	USA.	Qualitative (thematic analysis).	Individual interviews with participants. Focus groups with participants following this to solicit feedback on key findings from the individual interviews.	16 adults with intellectual and developmental disabilities.	Three themes were identified. Research designs: participants value research and have much to contribute; they prefer freely expressing their opinions and feelings with open‐ended questions; they want to be compensated for their time; and they want researchers to portray them in a positive light. Providing accommodations: accommodating and supporting the needs of participants so they can participate in research. Researcher‐participant interactions: the importance of feeling respected in interactions, and this leading to respect for the researcher.
McDonald, Conroy, Kim, et al. (2016). Is safety in the eye of the beholder? Safeguards in research with adults with intellectual disability.	USA.	Mixed methods—qualitative (thematic analysis) and quantitative non‐randomised (cross‐sectional).	Structured interview survey (Project ETHICS). Focus on questions around safeguards for adults with intellectual disabilities participating in research regarding recruitment, decision‐making, and research participation, as well as views towards research and personal information.	101 adults with intellectual disabilities, 98 family members and close friends of adults with intellectual disabilities, 109 professionals who currently or have previously provided social services to adults with intellectual disabilities, 105 researchers, and 99 institutional review board (IRB) members.	Contrary to other groups, adults with intellectual disabilities rated a variety of recruitment strategies, including via service providers, researchers, and people with disabilities, as safe. They also perceived being directly involved in recruitment and making their own decisions to be among the safest safeguards. All groups perceived researchers talking to others before the adults with intellectual disabilities as significantly less safe. Adults with intellectual disabilities indicated that they would be more likely to participate in research than others thought they would be, particularly IRB members. Perceptions of safety were positively and moderately to strongly correlated with greater likelihood of participating. In open‐ended comments, participants noted the importance of additional contextual features, such as the nature of the study, and emphasised the importance of researchers being skilled in working with people with intellectual disabilities to promote respectful, accessible interactions.
McDonald et al. (2018). A quantitative study of attitudes towards the research participation of adults with intellectual disability: Do stakeholders agree?	USA.	Mixed methods—qualitative (thematic analysis) and quantitative non‐randomised (cross‐sectional).	Project ETHICS survey. Focus on questions about attitudes towards research participation of people with intellectual disabilities, as well as their past research experiences, general views on research, trust in researchers, and personal information.	101 adults with intellectual disabilities, 98 family members and close friends of adults with intellectual disabilities, 109 professionals who currently or have previously provided social services to adults with intellectual disabilities, 105 researchers, and 99 IRB members.	There was broad support for the importance of conducting research about adults with intellectual disabilities. There was more tempered support for their direct participation, particularly from adults with intellectual disabilities and IRB members. Generally, IRB members espoused somewhat less support for these ideas than most other groups. Adults with intellectual disabilities more strongly agreed that they can make up their own mind than family members and friends, and IRB members. Service providers involved in disability advocacy more strongly believed that adults with intellectual disabilities can make up their own minds than other service providers. IRB members involved in disability advocacy more strongly believed that it is very important that adults with intellectual disability take part in research and that individuals should be allowed to make up their own mind than other IRB members. In open‐ended comments, researchers and IRB members noted that the level of intellectual disability and nature of the study would impact views about the ability of potential participants to participate in research. Participants from all groups added that trust plays an important role in whether a person with intellectual disability desires support in making their participation decision and who they desire that support from.
McDonald et al. (2017). What's the harm? Harms in research with adults with intellectual disability.	USA.	Mixed methods—qualitative (thematic analysis) and quantitative non‐randomised (cross‐sectional).	Project ETHICS survey. Focus on questions about harms in research. Participants were asked to rate how harmful each of 13 events—exclusion from research, psychological and social events, and legal events—is for adults with intellectual disabilities and how likely they thought adults with intellectual disabilities would be to participate in research that might involve that harm. Attitudes towards research and personal information were also asked.	101 adults with intellectual disabilities, 98 family members and close friends of adults with intellectual disabilities, 109 professionals who currently or have previously provided social services to adults with intellectual disabilities, 105 researchers, and 99 IRB members.	Adults with intellectual disabilities and service providers see keeping a research participation opportunity from possible participants as more harmful than family and friends and IRB members. Adults with intellectual disabilities see it as more harmful than other groups if researchers need to report information to the authorities. According to open‐ended comments, other groups see this as harmful in the short‐term but more beneficial in the long‐term. All stakeholders believed that having someone else make the decision to participate is harmful. Adults with intellectual disabilities saw almost all events as less harmful than other groups and had moderate interest in participating in research. Other groups perceived greater fragility among adults with intellectual disabilities in their ability to successfully handle experiences. Adults with intellectual disabilities perceive some events as more harmful than others, including being treated as a label or condition rather than an adult or person, not maintaining confidentiality, and not ensuring the participation decision belongs to them.
McDonald et al. (2016). Is it worth it? Benefits in research with adults with intellectual disability.	USA.	Mixed methods—qualitative (thematic analysis) and quantitative non‐randomised (cross‐sectional).	Project ETHICS survey. Focus on questions about benefits in research. Participants were asked how important 11 experiences or outcomes of participation are for adults with intellectual disabilities or society, and how likely adults with intellectual disabilities would be to participate in research with each experience or outcome. Attitudes towards research and personal information were also asked.	101 adults with intellectual disabilities, 98 family members and close friends of adults with intellectual disabilities, 109 professionals who currently or have previously provided social services to adults with intellectual disabilities, 105 researchers and 99 IRB members.	Adults with intellectual disabilities highly value research benefits and are strongly interested in participating in research. Many adults with intellectual disabilities are motivated to help others with intellectual disabilities and to improve their quality of life. Concerns were expressed in open‐ended comments that adults with intellectual disabilities may not understand or value indirect benefits and that incentives would be coercive. This was challenged by the responses of adults with intellectual disabilities. Other stakeholders, particularly researchers and IRB members, may place less value than adults with intellectual disabilities on some direct benefits, including helping others, learning new things, meeting people, doing something new, and receiving incentives. Interest in research participation among adults with intellectual disabilities is often stronger than other groups predicted.
McDonald and Keys (2008). How the powerful decide: Access to research participation by those at the margins.	USA.	Quantitative non‐randomised (cross‐sectional).	Survey including research vignettes about fictitious research studies involving adults with or without intellectual disabilities, and that presented little or significant harm. Participants were asked questions related to capacity to consent, the degree of risk posed to participants, and the level of necessary safeguards or protections. Participants also answered questions about attitudes to research for people with intellectual disabilities.	116 IRB members, 114 researchers, and 30 individuals who were both IRB members and researchers.	Inclusion of adults with intellectual disabilities in research decreased perceptions of capacity and increased perceptions of risk and needed protections. Increasing harm also decreased decision makers' perceptions of capacity to consent and increased perceptions of risk and level of protection. Once decision makers' attitudes towards the research participation of adults with intellectual disabilities were taken into account, the main effects and interactions of disability and harm to access determinants disappeared.
McDonald et al. (2008). Gatekeepers of science: Attitudes towards the research participation of adults with intellectual disability.	USA.	Quantitative non‐randomised (cross‐sectional).	Survey including attitudes towards research participation of people with intellectual disabilities.	116 IRB members, 114 researchers, and 30 individuals who were both IRB members and researchers.	Specific attitudes towards research participation of adults with intellectual disabilities may be organised into three conceptual domains: choice and opportunity, helping in decision‐making (the tendency to protect adults with intellectual disabilities through assumptions of reduced autonomy), and beneficence (the need to maximise benefit, minimise harm, and promote participants' well‐being). Researchers and those with close relationships to individuals with disabilities had attitudes more consistent with disability‐rights principles. Some dimensions of global attitudes towards adults with intellectual disabilities predicted more specific attitudes towards their research participation.
McDonald et al. (2009). Including adults with intellectual disabilities in research: Scientists' perceptions of risks and protections.	USA.	Mixed methods—qualitative (grounded theory) and quantitative non‐randomised (cross‐sectional).	Survey including research vignettes about fictitious research studies involving adults with or without intellectual disabilities, and that presented little or significant harm. Participants were asked to identify specific risks and protections that participants might experience or need to secure their well‐being.	114 IRB members and 85 researchers.	More risks were noted by respondents when the hypothetical study was high harm than low harm, and IRB members identified more risks than researchers. Participants reported a need for more protections in high‐harm studies as well as studies that included adults with intellectual disabilities. There was more identification of psychological risk for participants with intellectual disabilities in both high and low harm vignettes.
McDonald et al. (2013). ‘You need to let your voice be heard’: Research participants' views on research.	USA.	Qualitative (thematic analysis).	Semi‐structured interviews asking participants about a recent research experience, including their experience, views on inclusion, recruitment, decision‐making, safeguards, risks, and benefits. Focus groups asking participants for reactions to findings, including their accuracy, relative importance, and completeness.	16 adults with intellectual and developmental disabilities. 12 of these 16 participants completed the focus groups.	Three main themes were identified. Improving quality of life: participants wanted to engage in research to make a difference; to benefit directly by learning new things, improving their lives, having new experiences, meeting new people, having something to do, and helping others; to participate in research with social value; and to be compensated for their time. Access to research participation: participants thought it was important for all people with intellectual disabilities to have a chance to participate in research; and valued various ways of recruitment, good quality materials which are brief, have visuals, and provide demonstrations, and the researcher explaining things to them. Trust: participants preferred to learn about research from people they trust; wanted to be in charge of their decision; valued having a supporter with them, but their decision still being their own. Trust in part related to feeling listened to.
McDonald et al. (2015). ‘You can't be cold and scientific’: Community views on ethical issues in intellectual disability research.	USA.	Qualitative (using multiple traditions including thematic analysis, grounded theory, and naturalistic inquiry).	Semi‐structured focus groups with participants about their general views on the participation of adults with intellectual disabilities in research, as well as perspectives on benefits, harms, safeguards, respect, and trust. Participants were also asked about views on how studies are conducted, including important topics to study, the role of people who support adults with intellectual disabilities, recruitment, decision‐making, and dissemination.	24 adults with intellectual disabilities (no prior experience of research participation), 21 people who provided social services to adults with intellectual disabilities, and 12 family members and/or close friends of adults with intellectual disabilities.	Four themes were identified. Inclusion in research (strong support for being included; decreased support for participation in relation to not being able to provide consent, sensitive topics, lack of accommodation and not knowing the researcher well), benefits of research participation, harms in research participation (concerns around understanding, being susceptible to undue influence and disclosures during the research process; difficulties trusting others and worries about being treated disrespectfully; researchers not having appropriate skills to work with people with intellectual disability) and safety in research (being treated with respect; being able to make their own decisions; accommodations and helping participants to feel comfortable; possible consultation with another person; getting to know the researcher beforehand; ensuring the participant can give informed consent).
Mulhall et al. (2020). Using a Delphi survey to gain an international consensus on the challenges of conducting trials with adults with intellectual disabilities.	UK.	Mixed‐methods—Delphi study. Qualitative (thematic‐content analysis approach) and quantitative descriptive (cross‐sectional).	Modified 3‐round Delphi study. Round 1: participants reported practical and methodological barriers or challenges when conducting RCTs for adults with intellectual disabilities. Stages 2 and 3: participants rated importance of items until consensus (> 70%) was achieved.	Internationally renowned intellectual disability RCT researchers. Round 1: 22, round 2: 21, round 3: 20.	56 items reached consensus in round 2 and 8 additional items reached consensus in round 3. Sections of barriers and challenges: trial planning, team planning, funding, ethics and consent, recruitment, participant factors, service factors, co‐participant factors, fidelity challenges, technical understanding, outcome measures, attitudes and perceptions.
Mulhall et al. (2021). Challenges to conducting randomised controlled trials with adults with intellectual disabilities: Experiences of international experts.	UK.	Qualitative (thematic analysis).	One‐to‐one semi‐structured interviews exploring methodological and practical challenges for conducting RCTs with adults with intellectual disabilities, covering: trial planning, funding, ethical approval and consent, recruitment, conducting the trial, analysis, and reporting.	12 researchers.	Three overarching themes. (1) Participant factors—varying ability levels and subsequent resource need; communicating complex RCT concepts; mandatory reporting following increased interaction with participants' home lives, relatives, and professional support staff; balancing representativeness and internal validity due to comorbidities. (2) Design factors—obtaining ethical approval and informed consent; recruitment via agencies and professionals; reliance on family and staff, and subsequent burden of this; funders limiting scope of trials. (3) Systems factors—gatekeeping and its influence on participation; staff turnover affecting recruitment, retention, and data collection; lack of technical understanding of RCTs; and randomisation making gatekeepers reluctant to support participation. A sub‐theme of attitudes and perceptions (adults with intellectual disabilities' research interest; funders promoting RCTs for this population; and the scientific community being unsure of benefits) influenced the three main themes and was influenced by ‘lack of understanding’.
Nicholson et al. (2013). Recruitment to intellectual disability research: A qualitative study.	UK.	Qualitative (framework approach).	Semi‐structured telephone interview with participants about their experiences during participant recruitment for another study, including successful and unsuccessful strategies.	10 intermediaries, including two members of the local intellectual disability services network, two doctors, two support workers, two team leaders/managers of support services, a manager of a resource centre, and a manager of a service user support group.	Seven themes were identified regarding recruitment: participant factors (anxiety about participating, not fully understanding the concept of research, busy schedules, and lack of interest in taking part); the research process (the value of the researcher meeting potential participants prior to recruitment, information packs tailored to potential participants); the importance of the research (the benefits and drawbacks of using the researcher's title and first name, and the research having a personal approach); the impact of previous experience (reduced interest after not having feedback of results from previous studies); families and carers (influence of their attitudes towards research); ‘active recruitment’ (actively approaching people, taking the time needed to explain the research, communication and advice about recruitment with intermediaries); motivators (importance of the research having a direct benefit for participants).
Robotham et al. (2011). Social validity of randomised controlled trials in health services research and intellectual disabilities: A qualitative exploration of stakeholder views.	UK.	Qualitative (framework approach, informed by ‘template analysis’).	Semi‐structured face‐to‐face interviews asking about RCTs, including processes, communication, and informed consent. These were following an RCT investigating a psychological intervention for challenging behaviour.	Six adults with mild intellectual disabilities, 11 paid carers, seven mothers of adults with intellectual disabilities, and 27 health and social care professionals who had been involved in a larger study by the researchers.	Most stakeholders viewed the methods underpinning RCTs and the need to conduct such research positively. Trial procedures, such as random allocation, were poorly understood within the service users and family carer groups. Stakeholders also raised concerns around random allocation, including it being perceived as unfair, not understanding what would be gained from participation in the research, and trials creating barriers to accessing services and certain treatments. Concerns were also raised about obtaining valid consent and the ethics of deciding on someone else's behalf. The desire for a multidisciplinary decision around participation in RCTs was raised by paid carers and professionals.
Strickler and Havercamp (2023). Evaluating an informed consent process design to improve inclusion of adults with intellectual disability in research.	USA.	Mixed methods—qualitative (grounded theory) and quantitative RCT.	Novel interactive teaching process (ITP) for informed consent consisting of three modes: mode A (conventional consent form), mode B (easy read), and mode C (conversational). Participants were randomised into one of two groups that differed only in the order of experimental conditions. Ability to provide informed consent was assessed. Participants were asked three questions to provide feedback on each mode.	21 adults with intellectual disabilities.	Only one participant demonstrated capacity to consent following ITP mode A. Only one of the remaining 20 participants achieved capacity to consent by the end of the process. Despite this, significant improvements were found in participants' comprehension of consent materials compared to baseline. Participants provided a strong endorsement of the two experimental approaches to presenting consent materials.
Swaine et al. (2011). Recruitment and consent of women with intellectual disabilities in a randomised control trial of a health promotion intervention.	USA.	Quantitative non‐randomised and descriptive (cross‐sectional).	Data collected about participant recruitment and consent as part of a larger study, including recruitment success rates and consent data for participants with and without a legal guardian who was authorised to make decisions on their behalf.	269 adult women with intellectual disabilities, including 90.6% with a mild to moderate intellectual disability.	There was a recruitment success rate of 75%. Enrolment rates were better for women who were their own guardians. Successful recruitment was due to participant payment, use of visual illustrations about the study, and genuine interest and motivation to participate. Reported anecdotal evidence for declining participation included: participants being embarrassed to talk about their bodies, feeling anxious to talk about cancer due to experience of people dying from it, and guardians not feeling that health classes were necessary, and concerns about teaching on sexual intercourse.

Reliability checks at the title and abstract screening stage resulted in 95% agreement between raters. Discrepancies were discussed and consensus was achieved, resulting in 100% agreement. For the full‐text screening stage, there was 100% agreement between raters.

### 3.2 Quality Appraisal

Quality appraisals were conducted using the MMAT critical appraisal tools for each paper included in the review (see Appendix A; Tables A1, A2, A3, A4, A5, A6). MMAT screening questions, analysing whether the research question was clear and whether data collected allowed the research question to be addressed, are not reported due to all papers meeting these criteria. In line with MMAT recommendations, each individual criterion rating is presented rather than an overall quality score to provide a more detailed representation of paper quality. The quality appraisal was completed independently by the first and second authors. During initial discussions, there was 81.51% consistency across ratings. After further discussion, 100% agreement was achieved.

### 3.3 Narrative Synthesis

Barriers and facilitators were reviewed according to SEM levels of influence. Intrapersonal factors reflected attitudes, beliefs, and experiences of adults with intellectual disabilities. Interpersonal factors captured family members' and unpaid carers' attitudes and beliefs, and their influence on participation. Institutional factors comprised organisations, including support services, researchers, and ethics committees, and their structures, rules, and practices. Community factors reflected relationships between organisations and other relevant groups, such as researcher‐service relationships and potential practical community‐level influences. Policy factors included laws, regulations, and guidance from ethics and funding bodies that influence research participation.

#### 3.3.1 Barriers

##### 3.3.1.1 Intrapersonal

Adults with intellectual disabilities reportedly did not participate in research due to a lack of perceived benefits (Hall et al. 2017; Robotham et al. 2011) and limited time (Hall et al. 2017; Nicholson et al. 2013). Concerns were raised about understanding what the research involved (Nicholson et al. 2013; Robotham et al. 2011), particularly for RCTs (Robotham et al. 2011), and whether they could provide consent (Robotham et al. 2011).

Barriers also related to research experiences, including dislike for being treated as a label or condition (McDonald et al. 2017), researchers needing to report information (McDonald et al. 2017), not making decisions themselves (McDonald et al. 2017), and not receiving feedback, making them feel unappreciated (Nicholson et al. 2013). Some individuals described anxiety around participation (Nicholson et al. 2013) due to perceived difficulties about participating and/or seeing the process as ‘scary’ (Nicholson et al. 2013, 650).

##### 3.3.1.2 Interpersonal

Family and carers expressed concerns around the fragility of adults with intellectual disabilities regarding research participation (Swaine et al. 2011). Specific worries included lack of understanding (McDonald et al. 2016), consent (McDonald et al. 2015; Robotham et al. 2011), sensitive topics (McDonald et al. 2015), harms and safety (McDonald et al. 2015, 2017), and coercive incentives (McDonald et al. 2016). In contrast to support services, researchers, and ethics committees, family and carers were less likely to agree that adults with intellectual disabilities can make up their minds regarding research participation (McDonald et al. 2018). Some also felt that some individuals are not interested in nor place value on research (Kyprianou et al. 2023; McDonald et al. 2016).

Family and carers described difficulty understanding what studies involved and how they would benefit participants, particularly for RCTs (Robotham et al. 2011). Some had less favourable attitudes towards research in general (Nicholson et al. 2013), including thinking the study was unnecessary (Swaine et al. 2011) or that the person with intellectual disability had nothing valuable to contribute (Ho et al. 2018).

###### 3.3.1.2.1 Quality Note

While papers highlighting the fragility of adults at this level are generally high quality, caution is warranted for some findings (McDonald et al. 2016; Swaine et al. 2011). It is unclear whether confounds were accounted for, meaning these results may differ had relevant confounds been considered, such as gender, prior research experience, or intellectual disability severity.

##### 3.3.1.3 Institutional

Barriers at this level include the shared and unique challenges of support services, researchers, and ethics committees.

###### 3.3.1.3.1 Perceived Fragility

Concerns about the fragility of adults with intellectual disabilities were frequently reported, including participants' ability to consent (Crook et al. 2016; Hall et al. 2017; McDonald et al. 2015, 2018; McDonald and Keys 2008; Robotham et al. 2011), understanding (Cook and Inglis 2009; Hall et al. 2017; McDonald et al. 2016; Mulhall et al. 2020, 2021), safety, harm or need for protection (Bishop et al. 2024; McDonald et al. 2008, 2009, 2015, 2017, 2018; McDonald, Conroy, Kim, et al. 2016; McDonald and Keys 2008), and risk of coercion (McDonald et al. 2016). Researchers felt concerned about the appropriateness of certain interventions for this population, such as talking therapies (Bishop et al. 2024). Support services described concerns about meeting research demands (Hall et al. 2017), discussing sensitive topics (McDonald et al. 2015), and their own influence on participant decision‐making (Nicholson et al. 2013). Ethics committees were more tentative and less supportive of direct participation (McDonald et al. 2018) and more likely to withhold research opportunities (McDonald et al. 2017).

####### 3.3.1.3.1.1 Quality Note

These papers are generally high quality, but similar caution should be taken when interpreting results from two papers (McDonald et al. 2016, 2009) due to similar concerns around confounds.

###### 3.3.1.3.2 Attitudes Towards Research

Institutions questioned whether adults with intellectual disabilities were interested in and/or valued research participation (McDonald et al. 2016; Mulhall et al. 2021). Some support staff reportedly lacked enthusiasm towards research (Crook et al. 2016). Similarly, researchers described uncertainty in the scientific community about the benefits of inclusion (Mulhall et al. 2021) and reduced motivation towards doing this (Bishop et al. 2024). Support services struggled to understand study aims and benefits, both for their services and participants (Crook et al. 2016; Hall et al. 2017; Mulhall et al. 2020; Robotham et al. 2011). Concerns around randomisation and control group assignment for RCTs meant they were less likely to support participation (Mulhall et al. 2020, 2021; Robotham et al. 2011).

###### 3.3.1.3.3 Resources, Time and Organisational Support

Support services experienced challenges in managing research demands alongside daily responsibilities (Corby and Sweeney 2019; Crook et al. 2016; Hall et al. 2017), especially in the context of high staff turnover (Mulhall et al. 2020, 2021) and organisational policies which felt unsupportive of research (Hall et al. 2017). Researchers experienced challenges adapting materials to different ability levels (Mulhall et al. 2020, 2021), finding time to do this (Bishop et al. 2024), and involving staff with relevant expertise to facilitate inclusion (Bishop et al. 2024).

###### 3.3.1.3.4 Research Design and Delivery Challenges

Researchers reported concerns around increased involvement with participants' lives, resulting in them potentially observing issues such as neglect that required reporting (Mulhall et al. 2021). RCTs were reportedly especially challenging because of population heterogeneity and comorbidities, which affected the balance between sample representativeness and internal validity (Mulhall et al. 2020, 2021).

###### 3.3.1.3.5 Consent

Gaining consent was a common institutional barrier, including ensuring understanding of research concepts and right to decline participation (Bishop et al. 2024; Cook and Inglis 2009, 2012; Mulhall et al. 2020), especially for RCTs (Mulhall et al. 2021). Even when using conversational, easy‐read, or visual adaptations, researchers still reported difficulties (Dye et al. 2007; Strickler and Havercamp 2023), especially when meeting participants without a guardian or carer present (Horner‐Johnson and Bailey 2013).

####### 3.3.1.3.5.1 Quality Note

Most papers in this section were of moderate to high quality. However, some (Dye et al. 2007; Horner‐Johnson and Bailey 2013; Strickler and Havercamp (2023)) did not meet full MMAT criteria due to studies not reporting how randomisation took place or risk of response bias, and groups not being comparable at baseline. Several studies were also published over 10 years ago, suggesting caution when generalising these findings to the current context.

##### 3.3.1.4 Community

Researchers frequently described challenges liaising with gatekeepers, including family, carers, and service staff. Gatekeepers described feeling too busy to support with the research (Corby and Sweeney 2019; Ho et al. 2018; Mulhall et al. 2020). These challenges resulted in researchers' concerns about becoming a burden or nuisance (Corby and Sweeney 2019; Mulhall et al. 2020, 2021). In some cases, concerns about the research led gatekeepers to withhold support, preventing participation (Mulhall et al. 2020; Nicholson et al. 2013). This was particularly challenging given their influence on whether or not adults with intellectual disabilities participated in research (Bishop et al. 2024; Mulhall et al. 2020, 2021; Swaine et al. 2011).

##### 3.3.1.5 Policy

Policies were reported to restrict inclusion. Researchers described challenges securing adequate funding to adapt resources and have additional time to enable inclusion (Bishop et al. 2024; Mulhall et al. 2020, 2021). Researchers felt that funders did not believe research involving adults with intellectual disabilities is worth funding since it is less cost‐effective than other research (Mulhall et al. 2021). It was also described how ethics requirements regarding consent, despite attempting to protect individuals, were restrictive, subsequently preventing participation (Bishop et al. 2024).

#### 3.3.2 Facilitators

##### 3.3.2.1 Intrapersonal

Many adults with intellectual disabilities expressed enthusiasm regarding research participation, challenging views around disinterest. They reported positive attitudes towards research (Conroy et al. 2021; Crook et al. 2016; Frankena et al. 2018; Hall et al. 2017), valued it (McDonald et al. 2016), were interested in it (Swaine et al. 2011), and wanted to participate (Conroy et al. 2021; McDonald et al. 2016, 2013, 2015; Swaine et al. 2011). Kyprianou et al. (2023) found greater willingness to participate in psychosocial research than biomedical research, particularly research that could be completed at home. Younger adults (< 40) were more willing to partake than older adults.

These findings contradict reports that these individuals do not want to participate (Corby and Sweeney 2019; Nicholson et al. 2013). It is possible that the findings reflect a general divergence of attitudes that occurs in many populations, instead of it being the more predominant finding that adults with intellectual disabilities are uninterested in research. According to findings, particularly from papers directly recruiting these individuals, many are genuinely interested. While some papers were of lower quality due to concerns about sample representativeness (Kyprianou et al. 2023) and whether confounds were accounted for (McDonald et al. 2016), there remain several papers of high quality which demonstrate the same findings about attitudes.

Also contrary to the beliefs of others, adults with intellectual disabilities valued motivators and benefits to research participation (McDonald et al. 2013, 2015, 2016; Nicholson et al. 2013), including: payment or incentives (Crook et al. 2016; McDonald et al. 2016; Swaine et al. 2011), gaining knowledge (Lennox et al. 2005), helping others (Lennox et al. 2005), and receiving study results (Frankena et al. 2018). They preferred being recruited through familiar people, including services, people with disabilities, or researchers (McDonald, Conroy, Kim, et al. 2016). They felt that research is not as harmful as believed by others and viewed exclusion from research as more harmful than potential risks (McDonald et al. 2017).

A strong researcher‐participant relationship was another key facilitator, particularly when there was trust (Conroy et al. 2021; McDonald et al. 2018, 2013), respect (McDonald 2012), being seen as a person rather than a label (McDonald et al. 2017), and direct contact (Corby and Sweeney 2019). Flexible, tailored study design, including flexibility with when the study takes place (Corby and Sweeney 2019), adapted materials and relevant methods (Cook and Inglis 2009, 2012; Hall et al. 2017; McDonald 2012), accommodations (McDonald 2012), visual illustrations (Cook and Inglis 2009, 2012; Swaine et al. 2011), and direct involvement and opportunities to make their own recruitment decisions (Bishop et al. 2024; McDonald, Conroy, Kim, et al. 2016) further facilitated participation.

##### 3.3.2.2 Interpersonal

Family and carers valued direct involvement of adults with intellectual disabilities in research and them making their own decisions regarding recruitment (McDonald, Conroy, Kim, et al. 2016). However, this contradicts other findings that they doubt that these individuals can decide themselves (McDonald et al. 2018). Family and carers also believed in the importance of trust (McDonald et al. 2018). One paper illustrated that many carers were interested in hearing about future studies (Kyprianou et al. 2023). However, its sample was not representative, so may not reflect general carer interest.

##### 3.3.2.3 Institutional

Support services, researchers, and ethics committees all believed that adults with intellectual disabilities should have direct involvement with research and decide themselves about participation (McDonald, Conroy, Kim, et al. 2016), and that trust with the researcher was needed (McDonald et al. 2018). Researchers and ethics committees also valued supporting decision‐making, choice, and opportunity, with many researchers additionally holding attitudes consistent with disability rights (McDonald and Keys 2008). Many support staff held positive attitudes towards research (Crook et al. 2016) and highlighted the benefits of ‘active recruitment’—researchers taking the time needed to explain the research and following the process until they were satisfied they had completed their part in recruitment (Nicholson et al. 2013).

Facilitators regarding gaining consent included providing accessible and understandable information (Bishop et al. 2024; Cameron and Murphy 2007; Cook and Inglis 2009, 2012), meeting potential participants face‐to‐face to pick up on signals (Cameron and Murphy 2007), and providing opportunities to ask questions (Bishop et al. 2024). Having another person consent to participation also mitigated consent barriers (Bishop et al. 2024; Ho et al. 2018; Lennox et al. 2005). Additional strategies to enhance understanding included encouraging participants to repeat information in their own words, peer‐supported discussions about research and consent, a recursive approach to checking understanding, making the process enjoyable and intellectually stimulating, and using multiple learning approaches (Cook and Inglis 2009, 2012). Specialist training in intellectual disabilities further facilitated participation (Corby and Sweeney 2019; McDonald, Conroy, Kim, et al. 2016).

###### 3.3.2.3.1 Quality Note

Caution should be taken when interpreting these findings due to some papers (Bishop et al. 2024; Cameron and Murphy 2007; Corby and Sweeney 2019; Lennox et al. 2005) not meeting all MMAT criteria due to their not reporting on accounting for confounders, risk of nonresponse bias, how themes were derived from data, or the appropriateness of measures used to gain consent.

##### 3.3.2.4 Community

Support services valued increased researcher‐service liaisons, including identifying the most appropriate contact for recruitment, collaboratively discussing recruitment strategies, and ensuring staff can support participants, as well as support from management (Hall et al. 2017). Recognising the key role of staff in conducting research, including acknowledgement, thanks, or rewards, was also considered important (Hall et al. 2017). Services valued making multidisciplinary team decisions around service user participation in RCTs (Robotham et al. 2011). Services felt that researchers needed to be flexible when approaching services, including using multiple contact methods, contacting multiple organisations, developing relationships, and providing clear study information (Hall et al. 2017). Services were more willing to support studies that included adaptations and relevant methods (Hall et al. 2017) and engaged in ‘active recruitment’ whereby researchers discussed barriers and clarified staff roles (Nicholson et al. 2013).

###### 3.3.2.4.1 Quality Note

Most evidence for these facilitators, aside from general attitudes and beliefs, derives from Hall et al. (2017). Although this study met all MMAT criteria, replication across other services would provide more robust evidence of facilitators.

##### 3.3.2.5 Policy

None of the included papers highlighted facilitators at this level, highlighting a gap in the literature regarding how policies might encourage research participation by adults with intellectual disabilities.

Table 4 provides a summary of the key barriers and facilitators identified across the five SEM levels.

**TABLE 4:** Summary of barriers and facilitators identified.

SEM level	Key barriers	Key facilitators
Intrapersonal	Perceived lack of benefit; lack of time; understanding and consent concerns; anxiety; negative prior research experiences	Interest in and positive attitudes towards research; incentives; perceived benefits of participation; strong researcher‐participant relationships; tailored approaches
Interpersonal	Family/carer concerns about participant fragility, understanding, consent, and harm; undervaluing participant contribution or interest; lack of understanding of the research	Direct involvement for participants; trust
Institutional	Perceived fragility; risk‐averse attitudes; perceived lack of interest; resource/time constraints; research design and delivery challenges; consent challenges; researcher expertise	Inclusive attitudes; trust; supported decision‐making; positive attitudes towards research; ‘active recruitment’; accessible materials; researcher training
Community	Liasing with gatekeepers; lack of time; organisational burden	Strong researcher‐service relationships; staff recognition; researcher flexibility; ‘active recruitment’
Policy	Restrictive ethics processes; limited funding	None identified in included studies (suggests evidence gap)

## 4 Discussion

### 4.1 Findings and Implications

This review investigated barriers and facilitators to participation in psychosocial research by adults with intellectual disabilities, using the SEM to identify individual and shared barriers and facilitators across levels of influence. Barriers clustered around avoiding risk, attitudes towards research and inclusion, stakeholder relationships, and practical issues for conducting research. Facilitators included positive research attitudes, supportive stakeholder relationships, and tailored, person‐centred approaches to research. Tailored, person‐centred approaches are consistent with UK guidance on supporting research participation by adults with intellectual disabilities, including reasonable adjustments, accessible information, and participant‐centred recruitment and consent processes (Carey and Giffiths 2016; Equality Act 2010; Department of Health 2005). Results suggest that participation is dependent on addressing barriers at all levels of influence, as a block at any stage can prevent involvement.

Many barriers and facilitators identified are in line with findings from systematic reviews investigating other populations, including patients participating in health research (Sheridan et al. 2020), pregnant women (Van der Zande et al. 2018), minority ethnic groups (George et al. 2014), and children participating in paediatric research (Nathe et al. 2023). Reported barriers across groups include anxiety about what is involved with research and potential risks, practical challenges, distrust, lack of choice, competing life demands, poor access to study information, and consent challenges. Similar facilitators included benefits for participation (including links to altruism), trust, convenient and supported participation processes, key stakeholders being supportive of research, having additional support and information regarding consent, and positive participant‐researcher relationships.

Previous reviews highlighted barriers and facilitators across levels of influence for research, not only at the participant level. Nathe et al. (2023) reported parental discomfort with consenting on behalf of their child, mirroring interpersonal level barriers in this review. Other reviews also noted the influence of families and services on participation (Sheridan et al. 2020; Van der Zande et al. 2018). Nevertheless, they focused mainly on participant‐level barriers and facilitators, often involving participants who could consent independently. In contrast, these findings demonstrate how research with adults with intellectual disabilities involves additional complexity, requiring consideration of multiple levels of influence and targeting each of these.

Results revealed conflicting views and beliefs between levels of influence which may contribute to difficulties in research participation for adults with intellectual disabilities. A common belief was that these individuals are not interested or motivated to participate in research and are unmotivated by incentives (e.g., Kyprianou et al. 2023; Mulhall et al. 2021). Nevertheless, results at the intrapersonal level challenged this, showing that many value research participation and the use of incentives (e.g., Crook et al. 2016; Swaine et al. 2011). Similarly, while concerns were raised by different stakeholders about ability to cope (e.g., McDonald et al. 2015, 2017), adults with intellectual disabilities reported less concern, emphasising instead the importance of having opportunities and support to participate (McDonald et al. 2017). These findings highlight the need to challenge pre‐existing views about disinterest and undervaluing incentives, and to recognise how perspectives differ between different levels of influence. Findings are also in line with the wider literature; adults with intellectual disabilities often report increased interest in opportunities, belief in their capabilities, and desire for autonomy, while family, carers, and support staff often assume disinterest or emphasise the need for support and protection (Chadwick 2022; DuBois et al. 2019; Stefánsdóttir et al. 2018).

Findings suggested that some individuals at the interpersonal level hold less favourable attitudes towards research participation by adults with intellectual disabilities compared to other stakeholder groups (Nicholson et al. 2013; Swaine et al. 2011). Addressing this barrier may involve working with them directly to improve research attitudes. This includes increasing understanding of research and its benefits, challenging views that adults with intellectual disabilities lack interest or are unable to make valuable contributions, and reassuring concerns around consent. Nevertheless, few facilitators were identified for family and carers, leaving limited evidence about what supports participation at this level. This gap is concerning given the pivotal role that family and carers play, particularly for individuals with more moderate to severe intellectual disabilities who require additional support to consent and/or participate. Further research is therefore needed to identify facilitators at the interpersonal level, particularly improving research attitudes and subsequently gaining families' and carers' support to encourage participation. This also aligns with recommendations from research investigating barriers and facilitators to research participation for other populations (Connell et al. 2001; Graham et al. 2015).

Themes around avoiding risk are in accordance with general literature around intellectual disabilities. Vulnerabilities of these individuals are continuously described, resulting in risk‐averse policies and practices which prioritise preventing harm over embracing potential benefits after engaging in actions involving risk (Seale et al. 2013). Despite recognising the need for risk‐taking, family, carers, and services also frequently adopt restricted approaches to risk (Alaszewski and Alaszewski 2002). These findings are in line with Prospect Theory (Kahneman and Tversky 1982) which describes how individuals are more influenced by the possibility of loss versus the possibility of equivalent gain. According to results, for adults with intellectual disabilities, perceived vulnerabilities and reduced understanding of research participation benefits by different levels of influence further reduce perceived gain of risk‐taking and increase perceived loss. Conversely, many individuals reported that exclusion is more harmful than participation risks. They also highlight the importance of person‐centred approaches, which incorporate individuals' expressions, preferences, and beliefs when making decisions (Santana et al. 2018). These findings demonstrate the dilemma faced by different levels of influence in balancing protection with autonomy for adults with intellectual disabilities. They also reinforce the importance of following existing guidance on supporting research participation; applying these recommendations can help researchers design studies that align with the safeguards and protections set in place by ethics committees.

Lastly, while at the institutional level, it was acknowledged that researchers can facilitate participation through the use of inclusive and adapted methods, barriers related to time and expertise constraints were also identified (Bishop et al. 2024). These findings reflect a common conflict experienced by researchers, who may wish to be more inclusive but lack the time, resources, knowledge, or training required to do so, as identified in other reviews (Mulhall et al. 2018; Shariq et al. 2023). Notably, researchers operate within diverse disciplinary traditions, methodological approaches, and academic cultures, which shape understandings of consent, risk, and inclusion. Assumptions that researchers possess the competencies needed to design accessible materials and effectively engage adults with intellectual disabilities may therefore act as an additional and important institutional‐level barrier to participation.

Based on the results of the study, the following recommendations can be made:

1. Strengthen relationships and communication between researchers, participants, family, carers, and support staff by increasing understanding of research aims, processes, and value, while challenging assumptions that adults with intellectual disabilities lack interest or capacity to participate. Similar assumptions among research ethics committees should also be challenged.
2. Increase collaboration and flexibility with family, carers, and support staff by using various communication methods, holding information sessions, and engaging in collaborative planning to reduce research demands. Researchers should also liaise with service leads and managers to identify ways of supporting staff capacity to facilitate participation, including recognising and valuing their contribution and highlighting the importance and potential benefits of research for staff and the people they support.
3. Provide targeted training for researchers and ethics committees in intellectual disability awareness, including supported decision‐making procedures, adapting consent and study materials using easy‐read and visual formats, recognising gatekeeping dynamics in recruitment, and managing risk‐averse ethics processes.
4. Adopt flexible and person‐centred study designs. This includes accommodating preferences for location and timing, using accessible materials and visual supports, and directly involving adults with intellectual disabilities in all stages of the research process where possible.
5. Follow inclusive consent procedures. This includes supported decision‐making, using accessible and understandable information, face‐to‐face meetings, repeating key information, using multiple learning approaches, and using peer support where appropriate.
6. Use appropriate incentives for participation, explain research benefits (including relevance of research to daily life and contributions to supporting others with intellectual disabilities as well as those supporting them), and provide accessible feedback on study results to participants, family, carers, and support staff.
7. Encourage funders, institutions, and ethics committees to allocate time, training, and resources for inclusive practices.

### 4.2 Limitations and Future Research

It is likely that many papers in this review had a degree of response bias, particularly at the intrapersonal level. This is because many studies included adults with intellectual disabilities who had already agreed to participate in research. While these individuals still highlighted barriers, they were still able to participate. Therefore, findings may not fully reflect the general population of adults with intellectual disabilities. Furthermore, almost half of the included papers involved individuals with mild to moderate intellectual disabilities, with limited clarity about the remaining papers. Therefore, barriers and facilitators identified may be specific to this group, raising questions about generalisability to those with more severe intellectual disabilities. This may also explain discrepancies between research beliefs and attitudes of adults with intellectual disabilities and stakeholders from other levels of influence who may consider the broader spectrum of severity, although this cannot be evidenced by the findings. Greater understanding of participants' disability severity would better inform understanding of barriers and facilitators to research participation.

Although included papers addressed multiple SEM levels, some outer‐layer influences, particularly the political level, were only lightly represented. Although challenges related to funding and ethics were highlighted, perspectives and influences of other key stakeholders at this level—funding bodies, policymakers, journal editors, commissioners, and the wider academic community—are missing. Their absence represents a significant gap in research, given the power they hold to enable or constrain research, and the perceived influence of this by individuals from other levels of influence.

Wider cultural, social, and political factors were also underrepresented. Research has demonstrated cultural differences in attitudes towards intellectual disabilities (Cornoldi et al. 2018; Scior et al. 2010) as well as policies on disability rights and inclusion which are likely to influence research participation. Therefore, it is important for future research to directly explore these influences, including cross‐cultural differences in the inclusion of people with intellectual disabilities in research, and identifying the policy, organisational, and cultural factors that facilitate or constrain inclusive research practices across cultures.

While papers investigating the experience of adults with intellectual disabilities as co‐researchers were excluded from this review, those investigating the potential influence of co‐researchers with intellectual disabilities on participation were relevant. Despite this, none of the included papers addressed this. Given the increasing emphasis placed on co‐production within this field, this highlights an important gap in the literature; future research should investigate whether and how co‐researcher involvement facilitates recruitment and inclusion of adults with intellectual disabilities as research participants.

Lastly, over a third of included papers had McDonald as the first or second author. While many of these papers were of high quality, according to MMAT quality appraisal, many used similar methodologies and also appeared to report on results from studies investigating the same participants. Although other papers reported similar barriers and facilitators, the dominance of the McDonald studies raises questions about the generalisability of findings, particularly for institutional and community levels which reflect ethics committee and researcher perspectives. There is therefore a risk that some identified barriers and facilitators are overrepresented.

## 5 Conclusion

This systematic review investigated barriers and facilitators to participation in psychosocial research by adults with intellectual disabilities from a wide range of empirical evidence which utilises different methodologies and investigates different levels of influence involved in research. The review highlights both shared and level‐specific barriers and facilitators. Contradictions between levels of influence are highlighted, such as differing research beliefs and attitudes, views around harms in research, and the use of incentives, demonstrating an important implication for researchers to consider when designing and conducting studies involving adults with intellectual disabilities. This review also demonstrates the additional complexity and challenges regarding research participation for adults with intellectual disabilities; it suggests the need to target different levels of influence, utilising different strategies to improve recruitment and subsequent research participation.

## Funding

The authors have nothing to report.

## Conflicts of Interest

The authors declare no conflicts of interest.

## Data Availability

No new data were generated or analysed in this study. All data used in this systematic review are from published literature cited in the paper.

## Appendix A Quality Appraisal for Included Studies

**TABLE A1:** MMAT scores—first eight qualitative studies (listed alphabetically).

Methodology quality criteria	Bishop et al. (2024)	Cook and Inglis (2009)	Cook and Inglis (2012)	Corby and Sweeney (2019)	Crook et al. (2016)	Hall et al. (2017)	Ho et al. (2018)	McDonald (2012)	McDonald, Conroy, Kim, et al. (2016)	McDonald et al. (2017)	McDonald et al. (2018)
1.1. Is the qualitative approach appropriate to answer the research question?	Yes	Yes	Yes	Yes	Yes	Yes	Yes	Yes	Yes	Yes	Yes
1.2. Are the qualitative data collection methods adequate to address the research question?	Yes	Yes	Yes	No	Yes	Yes	Yes	Yes	Yes	Yes	Yes
1.3. Are the findings adequately derived from the data?	Yes	Yes	Yes	Can't tell	Yes	Yes	Can't tell	Yes	Yes	Yes	Yes
1.4. Is the interpretation of results sufficiently substantiated by data?	Yes	Yes	Yes	Yes	Yes	Yes	Can't tell	Yes	Yes	Yes	Yes
1.5. Is there coherence between qualitative data sources, collection, analysis and interpretation?	Yes	Yes	Yes	Yes	Yes	Yes	Can't tell	Yes	Yes	Yes	Yes

**TABLE A2:** MMAT scores—last eight qualitative studies (listed alphabetically).

Methodology quality criteria	McDonald et al. (2016)	McDonald et al. (2009)	McDonald et al. (2013)	McDonald et al. (2015)	Mulhall et al. (2020)	Mulhall et al. (2021)	Nicholson et al. (2013)	Robotham et al. (2011)	Strickler and Havercamp (2023)
1.1. Is the qualitative approach appropriate to answer the research question?	Yes	Yes	Yes	Yes	Yes	Yes	Yes	Yes	Yes
1.2. Are the qualitative data collection methods adequate to address the research question?	Yes	Yes	Yes	Yes	Yes	Yes	Yes	Yes	Yes
1.3. Are the findings adequately derived from the data?	Can't tell	Yes	Yes	Yes	Yes	Yes	Yes	Yes	Yes
1.4. Is the interpretation of results sufficiently substantiated by data?	Can't tell	Yes	Yes	Yes	Yes	Yes	Yes	Yes	Yes
1.5. Is there coherence between qualitative data sources, collection, analysis and interpretation?	Can't tell	Can't tell	Yes	Yes	Yes	Yes	Yes	Yes	Yes

**TABLE A3:** MMAT scores—quantitative randomised controlled trials.

Methodology quality criteria	Dye et al. (2007)	Strickler and Havercamp (2023)
2.1. Is randomisation appropriately performed?	Can't tell	Can't tell
2.2. Are the groups comparable at baseline?	Yes	No
2.3. Are there complete outcome data?	Yes	Yes
2.4. Are outcome assessors blinded to the intervention provided?	Can't tell	Yes
2.5. Did the participants adhere to the assigned intervention?	Yes	Yes

**TABLE A4:** MMAT scores—quantitative non‐randomised studies.

Methodology quality criteria	Cameron and Murphy (2007)	Conroy et al. (2021)	Kyprianou et al. (2023)	Lennox et al. (2005)	McDonald, Conroy, Kim, et al. (2016)	McDonald et al. (2018)	McDonald et al. (2017)	McDonald et al. (2016)	McDonald and Keys (2008)	McDonald et al. (2008)	McDonald et al. (2009)	Swaine et al. (2011)
3.1. Are the participants representative of the target population?	Can't tell	Yes	No	Yes	Yes	Yes	Yes	Yes	Yes	Yes	Yes	Yes
3.2. Are the measures appropriate regarding both the outcome and intervention (or exposure)?	Can't tell	Yes	Yes	Yes	Yes	Yes	Yes	Yes	Yes	Yes	Yes	Yes
3.3. Are there complete outcome data?	Yes	Yes	Yes	Yes	Yes	Yes	Yes	Yes	Yes	Yes	Yes	Yes
3.4. Are the confounders accounted for in the design and analysis?	Can't tell	Yes	Yes	Can't tell	Yes	Yes	Yes	Can't tell	Yes	Yes	Can't tell	Can't tell
3.5. During the study period, is the intervention administered (or exposure occurred) as intended?	Yes	Yes	Yes	Yes	Yes	Yes	Yes	Yes	Yes	Yes	Yes	Yes

**TABLE A5:** MMAT scores—quantitative descriptive studies.

Methodology quality criteria	Bishop et al. (2024)	Cameron and Murphy (2007)	Conroy et al. (2021)	Corby and Sweeney (2019)	Frankena et al. (2018)	Horner‐Johnson and Bailey (2013)	Kyprianou et al. (2023)	Lennox et al. (2005)	Mulhall et al. (2020)	Swaine et al. (2011)	Ho et al. (2018)
4.1. Is the sampling strategy relevant to address the research question?	Yes	Yes	Yes	Yes	Yes	Yes	Yes	Yes	No	Yes	Yes
4.2. Is the sample representative of the target population?	Yes	Can't tell	Yes	Yes	Yes	Yes	No	Yes	No	Yes	Yes
4.3. Are the measurements appropriate?	Yes	Can't tell	Yes	Yes	Yes	Yes	Yes	Yes	Yes	Yes	Yes
4.4. Is the risk of nonresponse bias low?	Can't tell	Can't tell	Can't tell	Can't tell	Can't tell	Can't tell	Can't tell	Can't tell	Can't tell	Can't tell	Can't tell
4.5. Is the statistical analysis appropriate to answer the research question?	Yes	Yes	Yes	Yes	Yes	Yes	Yes	Yes	Yes	Yes	Yes

**TABLE A6:** MMAT scores—mixed methods studies—first five studies (listed alphabetically).

Methodology quality criteria	Bishop et al. (2024)	Corby and Sweeney (2019)	Ho et al. (2018)	McDonald, Conroy, Kim, et al. (2016)	McDonald et al. (2018)	McDonald et al. (2016)	McDonald et al. (2009)	Mulhall et al. (2020)	Strickler and Havercamp (2023)	McDonald et al. (2017)
5.1. Is there an adequate rationale for using a mixed methods design to address the research question?	Can't tell	No	Yes	Yes	Can't tell	Yes	Yes	Yes	Can't tell	Yes
5.2. Are the different components of the study effectively integrated to answer the research question?	Yes	Yes	Yes	Yes	Yes	Can't tell	Yes	Yes	Yes	Yes
5.3. Are the outputs of the integration of qualitative and quantitative components adequately interpreted?	Yes	Yes	Yes	Yes	Yes	No	Yes	Yes	Yes	Yes
5.4. Are divergences and inconsistencies between quantitative and qualitative results adequately addressed?	No	Can't tell	Yes	Yes	Yes	Can't tell	Yes	Yes	Yes	Yes
5.5. Do the different components of the study adhere to the quality criteria of each tradition of the methods involved?	Can't tell	No	No	Yes	Yes	No	No	No	No	Yes

## References

[jar70267-bib-0001] Alaszewski, A. , and H. Alaszewski . 2002. “Towards the Creative Management of Risk: Perceptions, Practices and Policies.” British Journal of Learning Disabilities 30: 56–62. 10.1046/j.1468-3156.2001.00153.x.

[jar70267-bib-0002] Association of Medical Research Charities . 2022. “Shared Commitment to Public Involvement.” https://www.amrc.org.uk/shared‐commitment‐to‐public‐involvement#:~:text=In%20March%202022%2C%20AMRC%2C%20in%20collaboration%20with%20funders%2C,up%20standards%20in%20health%20and%20social%20care%20research.

[jar70267-bib-0003] Bishop, R. , R. Laugharne , N. Shaw , et al. 2024. “The Inclusion of Adults With Intellectual Disabilities in Health Research—Challenges, Barriers and Opportunities: A Mixed‐Methods Study Among Stakeholders in England.” Journal of Intellectual Disability Research 68, no. 2: 140–149. 10.1111/jir.13097. 37815212

[jar70267-bib-0004] Bronfenbrenner, U. 1974. “Developmental Research, Public Policy, and the Ecology of Childhood.” Child Development 45, no. 1: 1–5. 10.2307/1127743.

[jar70267-bib-0005] Brooker, K. , K. van Dooren , C. H. Tseng , L. McPherson , N. Lennox , and R. Ware . 2015. “Out of Sight, Out of Mind? The Inclusion and Identification of People With Intellectual Disability in Public Health Research.” Perspectives in Public Health 135, no. 4: 204–211. 10.1177/175791391455258. 25381305

[jar70267-bib-0006] Cameron, L. , and J. Murphy . 2007. “Obtaining Consent to Participate in Research: The Issue Involved in Including People With a Range of Learning and Communication Disabilities.” British Journal of Learning Disabilities 35, no. 2: 113–120. 10.1111/j.1468-3156.2006.00404.x.

[jar70267-bib-0007] Carey, E. , and C. Giffiths . 2016. “The Impact of Irish Policy and Legislation on How Adults With Learning Disabilities Make Choices.” British Journal of Learning Disabilities 44, no. 2: 111–121. 10.1111/bld.12117.

[jar70267-bib-0008] Chadwick, D. D. 2022. “‘You Want to Know That You're Safe’: Experiences of Risk, Restriction and Resilience Online Among People With an Intellectual Disability.” Cyberpsychology: Journal of Psychosocial Research on Cyberspace 16, no. 3: 8. 10.5817/CP2022-3-8.

[jar70267-bib-0009] Cleaver, S. , H. Ouellette‐Kuntz , and A. Sakar . 2010. “Participation in Intellectual Disability Research: A Review of 20 Years of Studies.” Journal of Intellectual Disability Research 54, no. 3: 187–193. 10.1111/j.1365-2788.2010.01256.x. 20146739

[jar70267-bib-0010] Connell, C. M. , B. A. Shaw , S. B. Holmes , and N. L. Foster . 2001. “Caregivers' Attitudes Toward Their Family Members' Participation in Alzheimer Disease Research: Implications for Recruitment and Retention.” Alzheimer Disease and Associated Disorders 15, no. 3: 137–145. 10.1097/00002093-200107000-00005. 11522931

[jar70267-bib-0011] Conroy, N. E. , K. E. McDonald , R. S. Olick , and Project ETHICS Expert Panel Members . 2021. “A Survey Study of the Attitudes and Experiences of Adults With Intellectual Disability Regarding Participation in Research.” Journal of Intellectual Disability Research 65, no. 10: 941–948. 10.1111/jir.12877. 34369629 PMC8428784

[jar70267-bib-0012] Cook, T. , and P. Inglis . 2009. “Making Our Own Decisions: Researching the Process of ‘Being Informed’ With People With Learning Difficulties.” Research Ethics 5, no. 2: 55–64. 10.1177/174701610900500204.

[jar70267-bib-0013] Cook, T. , and P. Inglis . 2012. “Participatory Research With Men With Learning Disability: Informed Consent.” Tizard Learning Disability Review 17, no. 2: 92–101. 10.1108/13595471211218875.

[jar70267-bib-0014] Cooper, S. A. , G. McLean , B. Guthrie , et al. 2015. “Multiple Physical and Mental Health Comorbidity in Adults With Intellectual Disabilities: Population‐Based Cross‐Sectional Analysis.” BMC Family Practice 16: 1–11. 10.1186/s12875-015-0329-3. 26310664 PMC4551707

[jar70267-bib-0015] Corby, D. , and M. R. Sweeney . 2019. “Researchers' Experiences and Lessons Learned From Doing Mixed‐Methods Research With a Population With Intellectual Disabilities: Insights From the SOPHIE Study.” Journal of Intellectual Disabilities 23, no. 2: 250–265. 10.1177/1744629517747834. 29246083

[jar70267-bib-0016] Cornoldi, C. , A. Capaodieci , C. Coomer Diago , A. Miranda , and K. G. Shepherd . 2018. “Attitudes of Primary School Teachers in Three Western Countries Toward Learning Disabilities.” Journal of Learning Disabilities 51, no. 1: 43–54. 10.1177/0022219416678408. 27852876

[jar70267-bib-0017] Council for International Organizations of Medical Sciences (CIOMS) , and World Health Organization (WHO) . 2016. “International Ethical Guidelines for Health‐Related Research Involving Humans.” https://cioms.ch/wp‐content/uploads/2017/01/WEB‐CIOMS‐EthicalGuidelines.pdf. 40523065

[jar70267-bib-0018] Crook, B. , R. Tomlins , A. Bancroft , and L. Ogi . 2016. “‘So Often They Do Not Get Recruited’: Exploring Service User and Staff Perspectives on Participation in Learning Disability Research and the Barriers That Inhibit It.” British Journal of Learning Disabilities 44, no. 2: 130–137. 10.1111/bld.12120.

[jar70267-bib-0019] d'Abrera, J. C. , A. J. Holland , J. Landt , G. Stocks‐Gee , and S. H. Zaman . 2013. “A Neuroimaging Proof of Principle Study of Down's Syndrome and Dementia: Ethical and Methodological Challenges in Intrusive Research.” Journal of Intellectual Disability Research 57, no. 2: 105–118. 10.1111/j.1365-2788.2011.01495.x. 22044507

[jar70267-bib-0020] Dattilo, J. 2013. “Inclusive Leisure and Individuals With Intellectual Disability.” Inc 1, no. 1: 76–88. 10.1352/2326-6988-1.1.076.

[jar70267-bib-0021] Department of Health . 2005. “Mental Capacity Act.” https://www.legislation.gov.uk/ukpga/2005/9/contents.

[jar70267-bib-0022] Di Lorito, C. , A. Bosco , L. Birt , and A. Hassiotis . 2018. “Co‐Research With Adults With Intellectual Disability: A Systematic Review.” Journal of Applied Research in Intellectual Disabilities 31: 669–686. 10.1111/jar.12435. 29231285

[jar70267-bib-0023] DuBois, D. , R. Renwick , M. Chowdhury , S. Eisen , and D. Cameron . 2019. “Engagement in Community Life: Perspectives of Youths With Intellectual and Developmental Disabilities on Families' Roles.” Disability and Rehabilitation 42, no. 20: 2923–2934. 10.1080/09638288.2019.1576781. 30982357

[jar70267-bib-0024] Dye, L. , D. J. Hare , and S. Hendy . 2007. “Capacity of People With Intellectual Disability to Consent to Take Part in a Research Study.” Journal of Applied Research in Intellectual Disabilities 20, no. 2: 168–174. 10.1111/j.1468-3148.2006.00310.x.

[jar70267-bib-0025] Einfeld, S. L. , and B. J. Tonge . 1996. “Population Prevalence of Psychopathology in Children and Adolescents With Intellectual Disability: II Epidemiological Findings.” Journal of Intellectual Disability Research 40, no. 2: 99–109. 10.1046/j.1365-2788.1996.768768.x. 8731467

[jar70267-bib-0026] Elder, J. P. , L. Lytle , J. F. Sallis , et al. 2007. “A Description of the Social‐Ecological Framework Used in the Trial of Activity for Adolescent Girls (TAAG).” Health Education Research 22, no. 2: 155–165. 10.1093/her/cyl059. 16855014 PMC2764407

[jar70267-bib-0027] Equality Act . 2010. “Equality Act 2010, c. 15.” https://www.legislation.gov.uk/ukpga/2010/15/contents/enacted.

[jar70267-bib-0028] Feldman, M. A. , J. Bossett , C. Collet , and P. Burnham‐Riosa . 2014. “Where Are Persons With Intellectual Disabilities in Medical Research? A Survey of Published Clinical Trials.” Journal of Intellectual Disability Research 58, no. 9: 800–809. 10.1111/jir.12091. 24001184

[jar70267-bib-0029] Frankena, T. K. , J. Nalldenberg , N. Bekkema , H. J. van Schrojenstein Lantman‐de Valk , M. Cardol , and G. Leusink . 2018. “An Exploration of the Participation of People With Intellectual Disabilities in Research—A Structured Interview Survey.” Journal of Applied Research in Intellectual Disabilities 31, no. 5: 942–947. 10.1111/jar.12453. 29608236

[jar70267-bib-0030] George, S. , N. Duran , and K. Norris . 2014. “A Systematic Review of Barriers and Facilitators to Minority Research Participation Among African Americans, Latinos, Asian Americans, and Pacific Islanders.” American Journal of Public Health 104, no. 2: e16–e31. 10.2105/AJPH.2013.301706. PMC3935672 24328648

[jar70267-bib-0031] Gilmore, L. , and M. Cuskelly . 2014. “Vulnerability to Loneliness in People With Intellectual Disability: An Explanatory Model.” Journal of Policy and Practice in Intellectual Disabilities 11, no. 3: 192–199. 10.1111/jppi.12089.

[jar70267-bib-0032] Graham, A. , M. A. Powell , and N. Taylor . 2015. “Ethical Research Involving Children: Encouraging Reflexive Engagement in Research With Children and Young People.” Children & Society 295: 331–343. 10.1111/chso.12089.

[jar70267-bib-0033] Hall, N. , M. A. Durand , and S. E. Mengoni . 2017. “‘…Their Opinions Mean Something’: Care Staff's Attitudes to Health Research Involving People With Intellectual Disabilities.” British Journal of Learning Disabilities 45, no. 3: 198–207. 10.1111/bld.12195.

[jar70267-bib-0035] Ho, P. , J. Downs , C. Bulsara , S. Patman , and A. M. Hill . 2018. “Addressing Challenges in Gaining Informed Consent for a Research Study Investigating Falls in People With Intellectual Disability.” British Journal of Learning Disabilities 46, no. 2: 92–100. 10.1111/bld.12217.

[jar70267-bib-0036] Hong, Q. N. , S. Fàbregues , G. Bartlett , et al. 2018. “The Mixed Methods Appraisal Tool (MMAT) Version 2018 for Information Professionals and Researchers.” Education for Information 34, no. 4: 285–291. 10.3233/EFI-180221.

[jar70267-bib-0037] Horner‐Johnson, W. , and D. Bailey . 2013. “Assessing Understanding and Obtaining Consent From Adults With Intellectual Disabilities for a Health Promotion Study.” Journal of Policy and Practice in Intellectual Disabilities 10, no. 3: 260–265. 10.1111/jppi.12048. PMC3821759 24223054

[jar70267-bib-0038] Iacono, T. , and V. Murray . 2003. “Issues of Informed Consent in Conducting Medical Research Involving People With Intellectual Disability.” Journal of Applied Research in Intellectual Disabilities 16: 41–51. 10.1046/j.1468-3148.2003.00141.x.

[jar70267-bib-0039] Kahneman, D. , and A. Tversky . 1982. “The Psychology of Preferences.” Scientific American 246, no. 1: 160–173.

[jar70267-bib-0040] Karam, S. M. , M. Riegel , S. L. Segal , et al. 2015. “Genetic Causes of Intellectual Disability in a Birth Cohort: A Population‐Based Study.” American Journal of Medical Genetics Part A 167, no. 6: 1204–1214. 10.1002/ajmg.a.37011. 25728503 PMC4863139

[jar70267-bib-0041] Khan, M. , H. K. Brown , Y. Lunsky , et al. 2021. “A Socio‐Ecological Approach to Understanding the Perinatal Care Experiences of People With Intellectual and/or Developmental Disabilities in Ontario, Canada.” Women's Health Issues 31, no. 6: 550–559. 10.1016/j.whi.2021.08.002. 34556400 PMC8595790

[jar70267-bib-0042] Kinnear, D. , J. Morrison , L. Allan , A. Henderson , E. Smiley , and S. A. Cooper . 2018. “Prevalence of Physical Conditions and Multimorbidity in a Cohort of Adults With Intellectual Disabilities With and Without Down Syndrome: Cross‐Sectional Study.” BMJ Open 8, no. 2: e018292. 10.1136/bmjopen-2017-018292. PMC5829598 29431619

[jar70267-bib-0043] Kyprianou, N. , J. Hendrix , H. Hillerstrom , R. Grimm , A. M. Kirova , and E. Rubenstein . 2023. “Caregivers' Perception of Adults With Down Syndrome Willingness to Participate in Research.” Journal of Intellectual Disabilities Research 67, no. 4: 352–361. 10.1111/jir.12999. PMC11079918 36543755

[jar70267-bib-0044] Larentis, O. 2024. “Psychosocial Research: An Overview.” Journal of Medicinal and Organic Chemistry 7, no. 5: 257–258.

[jar70267-bib-0045] Lennox, N. , M. Taylor , T. Rey‐Conde , C. Bain , D. Purdie , and F. Boyle . 2005. “Beating the Barriers: Recruitment of People With a Learning Disability to Participate in Research.” Journal of Intellectual Disability Research 49, no. 4: 296–305. 10.1111/j.1365-2788.2005.00618.x. 15816817

[jar70267-bib-0046] Mazza, M. G. , A. Rossetti , G. Crespi , and M. Clerici . 2020. “Prevalence of Co‐Occurring Psychiatric Disorders in Adults and Adolescents With Intellectual Disability: A Systematic Review and Meta‐Analysis.” Journal of Applied Research in Intellectual Disabilities 33, no. 2: 126–138. 10.1111/jar.12654. 31430018

[jar70267-bib-0047] McDonald, K. E. 2012. “‘We Want Respect’: Adults With Intellectual and Developmental Disabilities Address Respect in Research.” American Journal on Intellectual and Developmental Disabilities 117, no. 4: 263–274. 10.1352/1944-7558-117.4.263. 22809073

[jar70267-bib-0048] McDonald, K. E. , N. E. Conroy , C. I. Kim , E. J. LoBracio , E. Prather , and R. S. Olick . 2016. “Is Safety in the Eye of the Beholder? Safeguards in Research With Adults With Intellectual Disability.” Journal of Empirical Research on Human Research Ethics 11, no. 5: 424–438. 10.1177/1556264616651182. 27307420 PMC5568911

[jar70267-bib-0049] McDonald, K. E. , N. E. Conroy , R. S. Olick , et al. 2018. “A Qualitative Study of Attitudes Toward the Research Participation of Adults With Intellectual Disability: Do Stakeholders Agree?” Disability and Health Journal 11, no. 3: 345–350. 10.1016/j.dhjo.2017.12.004. 29292211 PMC5999527

[jar70267-bib-0050] McDonald, K. E. , N. E. Conroy , R. S. Olick , and T. P. E. E. Panel . 2017. “What's the Harm? Harms in Research With Adults With Intellectual Disability.” American Journal of Intellectual and Developmental Disabilities 122, no. 1: 78–92. 10.1352/1944-7558-122.1.78. PMC5568892 28095059

[jar70267-bib-0051] McDonald, K. E. , N. E. Conroy , R. S. Olick , and Project ETHICS Expert Panel . 2016. “Is It Worth It? Benefits in Research With Adults With Intellectual Disability.” Intellectual and Developmental Disabilities 54, no. 6: 440–453. 10.1352/1934-9556-54.6.440. 27893316 PMC5568891

[jar70267-bib-0052] McDonald, K. E. , and C. B. Keys . 2008. “How the Powerful Decide: Access to Research Participation by Those at the Margins.” American Journal of Community Psychology 42: 79–93. 10.1007/s10464-008-9192-x. 18584318

[jar70267-bib-0053] McDonald, K. E. , C. B. Keys , and D. B. Henry . 2008. “Gatekeepers of Science: Attitudes Toward the Research Participation of Adults With Intellectual Disability.” American Journal of Mental Retardation 113, no. 6: 466–478. 10.1352/2008.113:466-478. 19127657

[jar70267-bib-0054] McDonald, K. E. , C. A. Kidney , S. L. Nelms , M. R. Parker , A. Kimmel , and C. B. Keys . 2009. “Including Adults With Intellectual Disabilities in Research: Scientists' Perceptions of Risks and Protections.” Journal of Policy and Practice in Intellectual Disabilities 6, no. 4: 244–252. 10.1111/j.1741-1130.2009.00225.x.

[jar70267-bib-0055] McDonald, K. E. , C. A. Kidney , and M. Patka . 2013. “‘You Need to Let Your Voice Be Heard’: Research Participants' Views on Research.” Journal of Intellectual Disability Research 57, no. 3: 216–225. 10.1111/j.1365-2788.2011.01527.x. 22292970

[jar70267-bib-0056] McDonald, K. E. , A. E. Schwartz , R. Dinerstein , R. Olick , and M. Sabatello . 2024. “Responsible Inclusion: A Systematic Review of Consent to Social‐Behavioral Research With Adults With Intellectual Disability in the US.” Disability and Health Journal 17: 101669. 10.1016/j.dhjo.2024.101669. 38960791 PMC13285379

[jar70267-bib-0057] McDonald, K. E. , A. E. Schwartz , and M. Sabatello . 2022. “Eligibility Criteria in NIH‐Funded Clinical Trials: Can Adults With Intellectual Disability Get in?” Disability and Health Journal 15, no. 4: 101368. 10.1016/j.dhjo.2022.101368. 36123292

[jar70267-bib-0058] McDonald, K. E. , N. M. Schwartz , C. M. Gibbons , and R. S. Olick . 2015. “‘You Can't Be Cold and Scientific’: Community Views on Ethical Issues in Intellectual Disability Research.” Journal of Empirical Research on Human Research Ethics 10, no. 2: 196–208. 10.1177/1556264615575512. 25769310 PMC4399491

[jar70267-bib-0059] McLennan, Y. , J. Polussa , F. Tassone , and R. Hagerman . 2011. “Fragile X Syndrome.” Current Genomics 12, no. 3: 216–224. 10.2174/138920211795677886. 22043169 PMC3137006

[jar70267-bib-0060] McLeroy, K. , D. L. Bibeau , A. Steckler , and K. Glanz . 1988. “An Ecology Perspective on Health Promotion Programs.” Health Education Quarterly 15, no. 4: 351–377. 10.1177/109019818801500401. 3068205

[jar70267-bib-0061] Merrells, J. , A. Buchanan , and R. Waters . 2019. “‘We Feel Left Out’: Experiences of Social Inclusion From the Perspective of Young Adults With Intellectual Disability.” Journal of Intellectual & Developmental Disability 44, no. 1: 13–22. 10.3109/13668250.2017.1310822.

[jar70267-bib-0062] Mulhall, P. , L. Taggart , V. Coates , and T. McAloon . 2020. “Using a Delphi Survey to Gain an International Consensus on the Challenges of Conducting Trials With Adults With Intellectual Disabilities.” Clinical Trials 17, no. 2: 138–146. 10.1177/1740774519887168. 31856601

[jar70267-bib-0063] Mulhall, P. , L. Taggart , V. Coates , T. McAloon , and A. Hassiotis . 2018. “A Systematic Review of the Methodological and Practical Challenges of Undertaking Randomised‐Controlled Trials With Cognitive Disability Populations.” Social Science & Medicine 200: 114–128. 10.1016/j.socscimed.2018.01.032. 29421458

[jar70267-bib-0064] Mulhall, P. , L. Taggart , T. McAloon , and V. Coates . 2021. “Challenges to Conducting Randomised Controlled Trials With Adults With Intellectual Disabilities: Experiences of International Experts.” Journal of Applied Research in Intellectual Disabilities 34, no. 3: 891–904. 10.1111/jar.12838. 33277777

[jar70267-bib-0065] Nathe, J. M. , T. T. Oskoui , and E. M. Weiss . 2023. “Parental Views of Facilitators and Barriers to Research Participation: Systematic Review.” Pediatrics 151, no. 1: e2022058067. 10.1542/peds.2022-058067. 36477217 PMC9808610

[jar70267-bib-0066] National Institute for Health and Care Research . 2020. “Improving Inclusion of Under‐Served Groups in Clinical Research: Guidance From the INCLUDE Project.” https://content.nihr.ac.uk/nihrdc/themedreview‐04326‐BCAHFA/Better‐Health_Care‐For‐FINALWEB.pdf.

[jar70267-bib-0067] Nicholson, L. , M. Colyer , and S. A. Cooper . 2013. “Recruitment to Intellectual Property Disability Research: A Qualitative Study.” Journal of Intellectual Disability Research 57, no. 7: 647–656. 10.1111/j.1365-2788.2012.01573.x. 22672134

[jar70267-bib-0068] Nind, M. , and H. Vinha . 2014. “Doing Research Inclusively: Bridges to Multiple Possibilities in Inclusive Research.” British Journal of Learning Disabilities 42: 102–109. 10.1111/bld.12013.

[jar70267-bib-0069] Oliver‐Africano, P. , S. Dickens , Z. Ahmed , et al. 2010. “Overcoming the Barriers Experienced in Conducting a Medication Trial in Adults With Aggressive Challenging Behaviour and Intellectual Disabilities.” Journal of Intellectual Disability Research 54, no. 1: 17–25. 10.1111/j.1365-2788.2009.01195.x. 19627427

[jar70267-bib-0070] Ouzzani, M. , H. Hammady , Z. Fedorowicz , and A. Elmagarmid . 2016. “Rayyan—A Web and Mobile App for Systematic Reviews.” Systematic Reviews 5, no. 1: 210. 10.1186/s13643-016-0384-4. 27919275 PMC5139140

[jar70267-bib-0071] Page, M. J. , J. E. McKenzie , P. M. Bossuyt , I. Boutron , T. C. Hoffmann , and J. McKenzie . 2021. “PRISMA 2020 Explanation and Elaboration: Updated Guidance and Exemplars for Reporting Systematic Reviews.” BMJ 372: n71. 10.1136/bmj.n71. 33781993 PMC8005925

[jar70267-bib-0072] Pardhan, S. , T. Sehmbi , R. Wijewickrama , H. Onumajuru , and M. P. Piyasena . 2025. “Barriers and Facilitators for Engaging Underrepresented Ethnic Minority Populations in Healthcare Research: An Umbrella Review.” International Journal for Equity in Health 24, no. 1: 1–16. 10.1186/s12939-025-02431-4. 40075407 PMC11905581

[jar70267-bib-0073] Popay, J. , H. Roberts , A. Sowden , et al. 2006. “Guidance on the Conduct of Narrative Synthesis in Systematic Reviews.” A Product From the ESRC Methods Programme, Version 1, no. 1: b92.

[jar70267-bib-0074] Robotham, D. , M. King , A. Canagasabey , S. Inchley‐Mort , and A. Hassiotis . 2011. “Social Validity of Randomised Controlled Trials in Health Services Research and Intellectual Disabilities: A Qualitative Exploration of Stakeholder Views.” Trials 12: 1–10. 21658215 10.1186/1745-6215-12-144 PMC3135548

[jar70267-bib-0075] Russell, G. , W. Mandy , D. Elliott , R. White , T. Pittwood , and T. Ford . 2019. “Selection Bias on Intellectual Disability in Autism Research: A Cross‐Sectional Review and Meta‐Analysis.” Molecular Autism 10, no. 1: 9. 30867896 10.1186/s13229-019-0260-x PMC6397505

[jar70267-bib-0076] Saldarriaga, W. , F. Tassone , L. Y. González‐Teshima , J. V. Forero‐Forero , S. Ayala‐Zapata , and R. Hagerman . 2014. “Fragile X Syndrome.” Colombia Médica 45, no. 4: 190–198. 25767309 PMC4350386

[jar70267-bib-0077] Salihu, H. M. , R. E. Wilson , L. M. King , P. J. Marty , and V. E. Whiteman . 2015. “Socio‐Ecological Model as a Framework for Overcoming Barriers and Challenges in Randomized Control Trials in Minority and Underserved Communities.” International Journal of MCH and AIDS 3, no. 1: 85–95. 27621990 PMC4948176

[jar70267-bib-0078] Santana, M. J. , K. Manalili , R. J. Jolley , S. Zelinsky , H. Quan , and M. Lu . 2018. “How to Practice Person‐Centred Care: A Conceptual Framework.” Health Expectations 21, no. 2: 429–440. 10.1111/hex.12640. 29151269 PMC5867327

[jar70267-bib-0079] Scior, K. , Y. Khan , A. McLoughlin , and J. Sheridan . 2010. “Public Attitudes Toward People With Intellectual Disabilities: A Cross‐Cultural Study.” Intellectual and Developmental Disabilities 48, no. 4: 278–289. 20722478 10.1352/1934-9556-48.4.278

[jar70267-bib-0080] Seale, J. , M. Nind , and B. R. Simmons . 2013. “Transforming Positive Risk‐Taking Practices: The Possibilities of Creativity and Resilience in Learning Disability Contexts.” Scandinavian Journal of Disability Research 15, no. 3: 233–248. 10.1080/15017419.2012.703967.

[jar70267-bib-0081] Shankar, R. , C. Rowe , A. Van Hoorn , et al. 2018. “Under Representation of People With Epilepsy and Intellectual Disability in Research.” PLoS One 13, no. 6: e0198261. 10.1371/journal.pone.0198261. 29927966 PMC6013187

[jar70267-bib-0082] Shariq, S. , A. M. Cardoso Pinto , S. S. Budhathoki , M. Miller , and S. Cro . 2023. “Barriers and Facilitators to the Recruitment of Disabled People to Clinical Trials: A Scoping Review.” Trials 24, no. 1: 171. 10.1186/s13063-023-07142-1. 36890505 PMC9994780

[jar70267-bib-0083] Shepherd, V. , F. Wood , R. Griffith , M. Sheehan , and K. Hood . 2019. “Protection by Exclusion? The (Lack of) Inclusion of Adults Who Lack Capacity to Consent to Research in Clinical Trials in the UK.” Trials 20: 1–8. 10.1186/s13063-019-3603-1. 31382999 PMC6683336

[jar70267-bib-0084] Sheridan, R. , J. Martin‐Kerry , J. Hudson , A. Parker , P. Bower , and P. Knapp . 2020. “Why Do Patients Take Part in Research? An Overview of Systematic Reviews of Psychosocial Barriers and Facilitators.” Trials 21: 1–18. 10.1186/s13063-020-4197-3. 32164790 PMC7069042

[jar70267-bib-0085] Sherman, S. L. , E. G. Allen , L. H. Bean , and S. B. Freeman . 2007. “Epidemiology of Down Syndrome.” Mental Retardation and Developmental Disabilities Research Reviews 13, no. 3: 221–227. 10.1002/mrdd.20157. 17910090

[jar70267-bib-0086] Simplican, S. C. , G. Leader , J. Kosciulek , and M. Leahy . 2015. “Defining Social Inclusion of People With Intellectual and Developmental Disabilities: An Ecological Model of Social Networks and Community Participation.” Research in Developmental Disabilities 38: 18–29. 10.1016/j.ridd.2014.10.008. 25543997

[jar70267-bib-0087] Spaul, S. W. , R. Hudson , C. Harvey , H. Macdonald , and J. Perez . 2020. “Exclusion Criteria: Learning Disability.” Lancet 395: e29. 32061300 10.1016/S0140-6736(20)30051-9

[jar70267-bib-0088] Stefánsdóttir, G. V. , K. Björnsdóttir , and Á. Stefánsdóttir . 2018. “Autonomy and People With Intellectual Disabilities Who Require More Intensive Support.” Scandinavian Journal of Disability Research 20, no. 1: 162–171. 10.16993/sjdr.21.

[jar70267-bib-0089] Stockton, M. B. , B. S. McClanahan , J. Q. Lanctot , R. C. Klesges , and B. M. Beech . 2012. “Identification of Facilitators and Barriers to Participation in Weight Gain Prevention Research by African American Girls.” Contemporary Clinical Trials 33: 38–45. 10.1016/j.cct.2011.08.010. 21924381

[jar70267-bib-0090] Strickler, J. G. , and S. M. Havercamp . 2023. “Evaluating an Informed Consent Process Design to Improve Inclusion of Adults With Intellectual Disability in Research.” Research in Developmental Disabilities 134: 104413. 10.1016/j.ridd.2022.104413. 36623399

[jar70267-bib-0091] Swaine, J. , S. L. Parish , K. Luken , and L. Atkins . 2011. “Recruitment and Consent of Women With Intellectual Disabilities in a Randomised Control Trial of a Health Promotion Intervention.” Journal of Intellectual Disability Research 55, no. 5: 474–483. 10.1111/j.1365-2788.2011.01399.x. 21385259

[jar70267-bib-0092] Sykes, K. , G. J. McGeechan , and E. L. Giles . 2024. “Exploring the Inequalities of Women With Learning Disabilities Deciding to Attend and Then Accessing Cervical and Beast Cancer Screening, Using the Sociol Ecological Model.” British Journal of Learning Disabilities 52: 538–548. 10.1111/bld.12587.

[jar70267-bib-0093] Taylor, S. , and J. McAvoy . 2015. “Researching the Psychosocial: An Introduction.” Qualitative Research in Psychology 12, no. 1: 1–7.

[jar70267-bib-0094] United Nations Convention on the Rights of Persons with Disabilities . 2006. “Convention on the Rights of Persons With Disabilities.” https://www.ohchr.org/en/instruments‐mechanisms/instruments/convention‐rights‐persons‐disabilities.

[jar70267-bib-0095] United Nations Human Rights Council . 2024. “Right to Participate in Science: Report of the Special Rapporteur in the Field of Cultural Rights.” https://docs.un.org/en/A/HRC/55/44.

[jar70267-bib-0096] Van der Zande, I. S. , R. van der Graaf , L. Hooft , and J. J. van Delden . 2018. “Facilitators and Barriers to Pregnant Women's Participation in Research: A Systematic Review.” Women and Birth 31, no. 5: 350–361. 10.1016/j.wombi.2017.12.009. 29373261

[jar70267-bib-0097] Wells, A. A. , and B. Zebrack . 2008. “Psychosocial Barriers Contributing to the Under‐Representation of Racial/Ethnic Minorities in Cancer Clinical Trials.” Social Work in Health Care 46, no. 2: 1–14. 10.1300/J010v46n02_01. 18192194

[jar70267-bib-0098] Whittle, E. L. , K. R. Fisher , S. Reppermund , and J. Trollor . 2019. “Access to Mental Health Services: The Experience of People With Intellectual Disabilities.” Journal of Applied Research in Intellectual Disabilities 32, no. 2: 368–379. 10.1111/jar.12533. 30306674

[jar70267-bib-0099] World Medical Association . 2024. “Declaration of Helsinki.” https://www.wma.net/policies‐post/wma‐declaration‐of‐helsinki/.

[jar70267-bib-0100] Yu, S. , T. Wang , T. Zhong , Y. Qian , and J. Qi . 2022. “Barriers and Facilitators to Physical Activity Participation Among Children and Adolescents With Intellectual Disabilities: A Scoping Review.” Healthcare 10: 233. 10.3390/healthcare10020233. 35206848 PMC8872190

